# Investigating pulmonary neuroendocrine cells in human respiratory diseases with airway models

**DOI:** 10.1242/dmm.050620

**Published:** 2024-05-30

**Authors:** Noah Candeli, Talya Dayton

**Affiliations:** European Molecular Biology Laboratory (EMBL) Barcelona, Tissue Biology and Disease Modelling, 08003, Barcelona, Spain

**Keywords:** Cancer, *In vitro* cultures, Pulmonary neuroendocrine cells, Respiratory diseases, Stem cells

## Abstract

Despite accounting for only ∼0.5% of the lung epithelium, pulmonary neuroendocrine cells (PNECs) appear to play an outsized role in respiratory health and disease. Increased PNEC numbers have been reported in a variety of respiratory diseases, including chronic obstructive pulmonary disease and asthma. Moreover, PNECs are the primary cell of origin for lung neuroendocrine cancers, which account for 25% of aggressive lung cancers. Recent research has highlighted the crucial roles of PNECs in lung physiology, including in chemosensing, regeneration and immune regulation. Yet, little is known about the direct impact of PNECs on respiratory diseases. In this Review, we summarise the current associations of PNECs with lung pathologies, focusing on how new experimental disease models, such as organoids derived from human pluripotent stem cells or tissue stem cells, can help us to better understand the contribution of PNECs to respiratory diseases.

## Teaser

**Summary:** This Review provides an overview of pulmonary neuroendocrine cells, with a focus on their role in lung pathologies and how *in vitro* models can be used to study them.

## Introduction

**Fig. 1.**
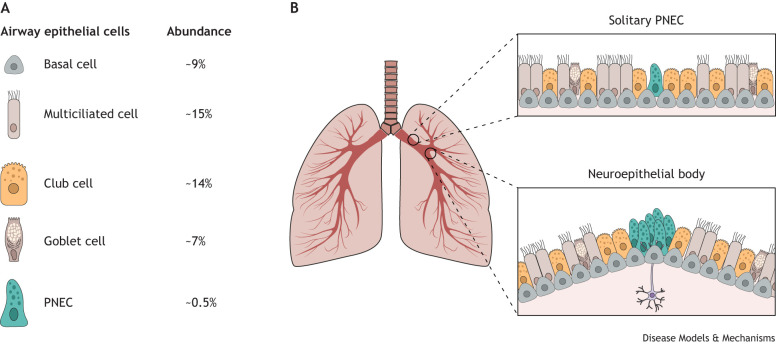
**Rarity of pulmonary neuroendocrine cells and their distribution in the airway.** (A) Representation of the abundance of different airway epithelial cell types, including pulmonary neuroendocrine cells (PNECs), relative to all lung epithelial cell types in both the proximal and the distal airways (calculated from Travaglini et al., 2020). (B) Spatial arrangement of PNECs in the airway epithelium as either solitary cells or aggregates called neuroepithelial bodies. Whereas neuroepithelial bodies are typically located at airway branch points and are frequently associated with nerves, solitary PNECs are found prominently in the trachea, as well as dispersed throughout the rest of the airways.

Pulmonary neuroendocrine cells (PNECs) are specialised cells that line the airways of the mammalian lung (Fig. 1). These cells are extremely rare compared to other lung epithelial cells and contribute to only ∼0.5% of the airway epithelium (Travaglini et al., 2020). They were first reported in the scientific literature in the late 1940s, when they were described as ‘Helle Zellen’ (‘bright cells’ in German), and were subsequently found to hold secretory, dense-core vesicles (see Glossary, Box 1) bearing bioactive compounds (Feyrter, 1954; Frohlich, 1949). PNECs are neuroendocrine cells resident in the lung, recognised to be critical airway sensors. Tissue-resident neuroendocrine cells of the pancreas and the intestine sense inputs from the environment and communicate with their surroundings and with other organ systems, such as the immune and nervous systems, to coordinate biological responses (Gunawardene et al., 2011; Röder et al., 2016). By definition, neuroendocrine cells are bifunctional epithelial cells that, in addition to having an endocrine function, have neuronal characteristics and express markers associated with the central nervous system. Canonical PNEC markers include achaete-scute family 1 [*ASCL1*, encoding a basic helix-loop-helix (bHLH) transcription factor] and chromogranin A (*CHGA*). Much like neurons, PNECs can process bioactive amines, such as serotonin (5-HT), and produce polypeptide hormones, such as bombesin (encoded by *GRP*) and calcitonin gene-related peptide 1 (CGRP-1, encoded by *CALCA*; hereafter CGRP) (Modlin et al., 2006; Garg et al., 2019). Their characteristic distribution, often found at branching points of the airway tree, is considered strategic for sensing environmental cues. They are thought to process information from the surrounding environment, orchestrating local (organ) and systemic (organismic) responses in homeostasis and disease. This cell population has recently emerged as being a multifaceted player involved in chemosensing, regeneration and immune regulation in the lung (Noguchi et al., 2020).

**Box 1. Glossary**

**Carcinoids:** slow-growing neuroendocrine cancers that are divided into two subcategories – typical carcinoids and atypical carcinoids (low- and intermediate-grade neuroendocrine tumours, respectively).

**Carcinomas:** fast-growing epithelial cancers. Poorly differentiated high-grade-tumours that have poor prognosis.

**Dense-core vesicles:** intracellular membrane-bound organelles found primarily in endocrine cells, containing a concentrated core of bioactive molecules such as neuropeptides, neurotransmitters and neurotrophins. These vesicles are involved in the storage and regulated release of signalling molecules via exocytosis.

**Hyperoxia:** a condition characterised by high oxygen concentration reaching the tissues in the body.

**Hypoxia:** a condition characterised by low oxygen concentration reaching the tissues in the body.

**Mitotic index:** ratio of the number of cancer cells undergoing mitosis to the total number of cancer cells.

**Mucosal type 2 response:** a physiological immune reaction characterised by the activation of type 2 immune pathways. This response typically involves the production of cytokines, which leads to the recruitment of eosinophils and mast cells and production of IgE antibodies. It plays a crucial role in defending against parasitic infections and allergic reactions and maintaining tissue homeostasis.

**Myoepithelial cells:** specialised epithelial cells found in glandular tissues, such as the mammary glands, salivary glands and sweat glands. They are located between the basement membrane and the secretory cells of the gland, and are characterised by their contractile properties, which enable them to assist in the expulsion of secretory products from exocrine glands.

**Naphthalene injury:** acute airway injury resulting from exposure to naphthalene, an aromatic hydrocarbon. It primarily affects club cells, which catalyze the conversion of naphthalene into the highly toxic naphthalene 1*R*,2*S*-oxide by cytochrome P450-2F2, resulting in necrosis.

**NE^stem^ cells:** rare subpopulation of fully differentiated pulmonary neuroendocrine cells (PNECs) that exhibit stem cell characteristics and populate neuroepithelial bodies (NEBs), typically comprising two to four cells per cluster within NEBs. Activated by injury, they are capable of renewal, dispersal, transit amplification and reprogramming.

**Primitive endodermal tube:** structure that forms during early embryonic development, precursor to the gastrointestinal tract. It initially forms as a simple tube-like structure, which eventually differentiates into the various parts of the digestive system (pharynx, esophagus, stomach, small intestine and large intestine).

**Submucosal glands:** glands located beneath the mucous membrane of various organs. They secrete mucus, enzymes and other substances into the lining of the organs, contributing to lubrication, protection and digestion.

**Tumourlets:** small, benign proliferations of neuroendocrine cells, typically measuring a few millimetres in size (<5 mm). They are usually found by chance in patients with prior lung disease requiring histology, are considered benign, and rarely metastasise. As detailed in the text, tumourlets are also found in patients with diffuse idiopathic PNEC hyperplasia (DIPNECH).

**Variant club cells (vCCs):** club cell-like population located around NEBs. These cells are unable to metabolise naphthalene, which renders them resistant to naphthalene-induced injury. Involved in the repair of the airway epithelium, they serve as transient-amplifying cells following injury.

Considering how rare they are in the lung epithelium, PNECs play an outsized role in respiratory disease. Increases in either PNEC numbers or in their secreted neuropeptides have been reported in a variety of respiratory diseases, including chronic obstructive pulmonary disease (COPD), asthma and cystic fibrosis (CF) (Johnson et al., 1988; Xu et al., 1999). PNECs also play a role in cancer. They are the primary cell of origin for small-cell lung cancer (SCLC), which accounts for 25% of aggressive lung cancers (Sutherland et al., 2011; Park et al., 2011). Likewise, PNECs are the presumed cells of origin for other subtypes of pulmonary neuroendocrine cancers, such as the less common large-cell neuroendocrine carcinoma (LCNEC) and lung neuroendocrine tumours or carcinoids (Box 1) (Rekhtman, 2022).

## PNEC development

**Fig. 2.**
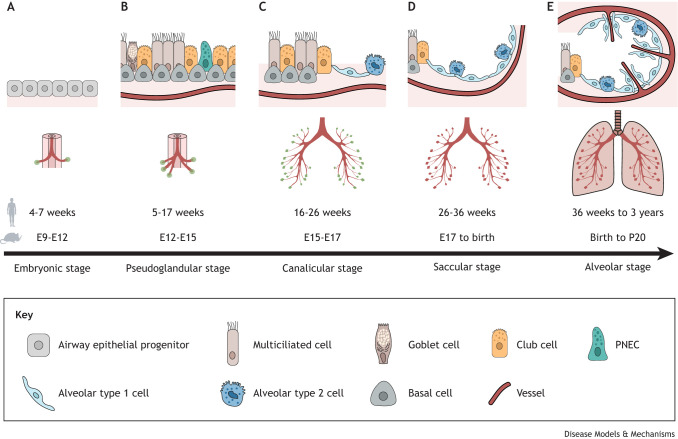
**Stages of lung development.** Schematic depicting lung morphology across the five different stages of lung development: embryonic, pseudoglandular, canalicular, saccular and alveolar. For each stage, the developmental period is indicated, for human in post-conception weeks and for mouse in embryonic days (‘E’) and postnatal days (‘P’). (A) During the embryonic phase, the primary left and right lung buds emerge from the anterior foregut endoderm. Each develops into an independent unit initiating branching and establishing the lung lobular structure. (B) During the pseudoglandular phase, the airway tree is formed and differentiation of the respiratory epithelium begins in the proximal airways. Airway epithelial progenitors in the lung bud tips (green) give rise to all lung epithelial cell types. Blood vessel development occurs concurrently with epithelial branching. (C) The canalicular phase is characterised by three additional rounds of epithelial branching, resulting in the formation of future alveolar regions. Existing airways continue to expand and distal epithelial tubes transition into thin-walled terminal saccules. Morphological signs of alveolar epithelial cell differentiation emerge and distal epithelial airspaces are vascularised by capillaries. (D) Saccular stage: coinciding with the cessation of branching morphogenesis, distal airspaces evolve into thin-walled terminal saccules. Saccules expand and become enveloped by capillaries, laying the foundation for gas exchange surfaces. (E) Alveolar stage: the alveolar formation process involves septal growth from saccular walls, subdividing distal saccules into alveoli and enhancing the surface area for gas exchange. Simultaneously, microvascular maturation occurs, ensuring complete coverage of each capillary by gas exchange surfaces. The concurrent increase in both the number and size of alveoli accounts for enlargement of the lung postnatally. PNEC, pulmonary neuroendocrine cells.

Studies in mice and experiments using *in vitro* human lung developmental models and RNA sequencing of human fetal lung tissue have helped to shed light on PNEC development. During mouse and human embryonic development, the lung evaginates from the primitive endodermal tube (Box 1), growing from a primary bud stage into a tree-like airway system via a process called branching morphogenesis. This resulting airway system contains a remarkably diverse population of epithelial cells (Fig. 2). The progeny of the cells in the bud tip give rise to all of the differentiated, canonical cell types of the proximal airways (basal cells, club cells, goblet cells, multiciliated cells and PNECs) and of the distal airways (alveolar type 1 and type 2 cells) (Rawlins et al., 2009). Several studies using mouse models have identified *Ascl1* and hairy and enhancer of split 1 (*Hes1*), members of the bHLH transcription factor family, as primary players in the regulation of PNEC differentiation from lung epithelial progenitors (Box 2) (Ito et al., 2000; Borges et al., 1997). PNECs are the first of these mature cell types to be specified in both the human and murine respiratory epithelium (Kuo and Krasnow, 2015; Sunday, 1996). Expression of *Ascl1* during embryonic mouse development is detected at embryonic day (E) 13.5 (Borges et al., 1997), whereas several studies observed PNECs in human fetal airways at 8-9 weeks of gestation (Sunday, 1996), before any other mature epithelial cell types appear in the lung.

**Box 2. PNEC progenitor differentiation**

The basic helix-loop-helix (bHLH) transcription factors encoded by *Ascl1* and *Hes1* are well-known, key regulators of neuronal cell commitment and differentiation in vertebrates (Lai et al., 2013), and play a similar role in pulmonary neuroendocrine cell (PNEC) development (Ito et al., 2000). *Ascl1* promotes the PNEC fate and is required for PNEC formation during development. There are no detectable PNECs in the lungs of *Ascl1*-null mice (Borges et al., 1997). Conversely, *Hes1* suppresses the PNEC fate, downregulating *Ascl1*. *Hes1*-deficient mice have higher *Ascl1* expression and an increased number of PNECs in their airways (Ito et al., 2000). *Hes1* is a Notch target gene, and Notch signalling is involved in determining PNEC cell fate in the fetal airway epithelium. Inactivation of the Notch receptors *Notch1*, *Notch2* and *Notch3* in mice results in abnormal increases in PNEC numbers and in neuroepithelial body size (Morimoto et al., 2012). A similar expansion of PNECs is observed in mice with double, but not single, inactivation of the Notch ligands *Dll1* and *Dll4*, which are selectively expressed in PNECs (Stupnikov et al., 2019). These results point to a Notch-mediated lateral inhibition model of PNEC fate selection. In this model, PNEC progenitors accumulate *Ascl1* expression and start to express Notch ligands (*Dll1* and *Dll4*), whereas in neighbouring cells, the activation of Notch receptors (*Notch1*, *Notch2* and *Notch3*) and their transcriptional target *Hes1* suppresses PNEC fate, leading to their differentiation to other secretory cell types such as club cells. Manipulation of Notch signalling through antagonists, such as DAPT {*N*-[*N*-(3,5-difluorophenacetyl)-L-alanyl]-*S*-phenylglycine *t*-butyl ester} or DBZ (dibenzazepine), has been successfully used in protocols to derive PNECs *in vitro* from human pluripotent stem cells (Chen et al., 2019; Hor et al., 2020; Konishi et al., 2016) and fetal airway progenitor cells (Miller et al., 2020).

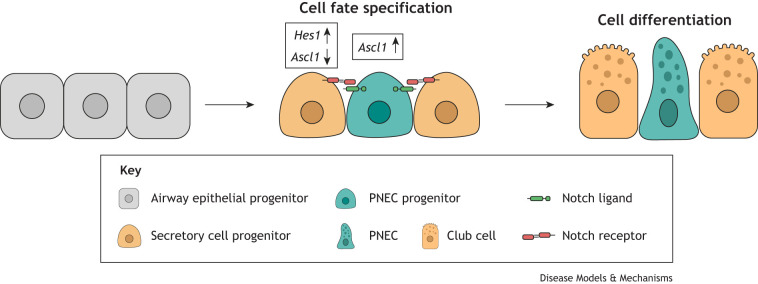

The multiple progenitor cell states that arise from the bud tip have not been entirely delineated. There is evidence from lineage tracing studies in mice that PNECs in the trachea and intrapulmonary airways (bronchi and bronchioles) can arise from cells that express the basal cell marker TP63 (also known as p63) at E9.5 (Yang et al., 2018a). Nevertheless, loss of these TP63^+^ cells in *Tp63* knockout mice does not influence the number of PNECs detected in the intrapulmonary airways at E18.5 (Yang et al., 2018a). Thus, TP63^+^ cells at E9.5 can give rise to PNECs, but TP63 expression is not required for PNEC formation in the developing mouse lung.

Song et al. (2012) provided evidence that intrapulmonary PNECs are derived from a CGRP-expressing precursor that can also give rise to alveolar cells early in mouse development. Lineage tracing of *CGRP*-expressing cells between E12.5 and E14.5 led to the labelling of intrapulmonary PNECs and of a small fraction of alveolar cells. By contrast, no alveolar cells were labelled when the same lineage tracing was performed at or after E15.5 (Song et al., 2012). Thus, during early mouse development, PNECs share a common progenitor with alveolar cells. In a recently published study, Conchola et al. (2023) identified a group of cells in fetal human airways that were enriched between 8 and 17 weeks post conception. These cells express a unique combination of genes, including *SCGB3A2*, *SFTPB* and *CFTR*, markers canonically associated with secretory, alveolar and ionocyte cell types, respectively. This population of cells was referred to as lower airway progenitors (LAP) and was shown to give rise to PNECs *in vitro* in lung organoid models (Conchola et al., 2023). It would be of interest to determine whether LAP cells can also give rise to alveolar cells and whether they represent similar progenitors to those observed by Song et al. (2012) in the mouse airways.

Collectively, these studies in mouse models and in cultured human fetal cells indicate that during lung development, PNECs can emerge from both basal cells and LAP cells. In the developing mouse lung, PNECs originating from basal cells appear to be limited to the tracheal region of the lung (Yang et al., 2018a). In the developing human lung, PNECs derived from LAP cells are likely to populate both intrapulmonary and tracheal airways, although this has not been explicitly shown. There is considerably less known about how PNECs are maintained throughout adulthood.

## PNECs in adult tissue

The question of whether PNECs arise throughout adulthood from neuroendocrine cell proliferation or postmitotic differentiation of non-neuroendocrine cells has not been resolved. Studies labelling PNECs in the murine airway have demonstrated a very low rate (1-2%) of PNEC proliferation (Song et al., 2012). Similarly, in humans, the proliferative fraction of PNECs observed from healthy patient autopsies is 1 to 2% (Boers et al., 1996). Such investigations, coupled with the understanding that PNECs have the capability to give rise to other PNECs in the context of airway injury (Ouadah et al., 2019), suggest that PNEC proliferation could contribute to PNEC maintenance during both injury and homeostasis.

Nevertheless, there is evidence from both mouse and human model systems that non-neuroendocrine cells, such as basal cells, can give rise to PNECs in adult airways. By combining *in vivo* lineage tracing with single-cell RNA sequencing (scRNA-seq), Montoro et al. (2018) demonstrated that in the mouse tracheal epithelium, PNECs are consistently and directly replenished by basal progenitor cells. Shivaraju et al. (2021) showed that after exposure to hypoxia (Box 1), lineage-traced basal cell progenitors in the murine trachea gave rise to PNECs. Based on pseudotime trajectory analysis of single-cell multiomics data of airway organoids derived from human distal lung tissue, it has been proposed that basal cells might undergo differentiation into PNECs also in adult human lungs (Lee et al., 2023). Lineage-tracing experiments in this human organoid model are needed to confirm the basal cell to PNEC trajectory proposed by pseudotime analysis.

Considering the diverse origins of PNECs during development, it is conceivable that distinct progenitor populations capable of giving rise to PNECs exist in the adult lung. Indeed, a progenitor population similar to fetal LAP cells has been identified in terminal respiratory bronchioles by two research groups (Kadur Lakshminarasimha Murthy et al., 2022; Basil et al., 2022). These progenitors have been demonstrated to serve as progenitors of alveolar cells. Given their resemblance to LAP progenitors, it would be intriguing to investigate, using *in vitro* models, whether this population could also generate PNECs.

## PNEC functions and neuroepithelial bodies

**Fig. 3.**
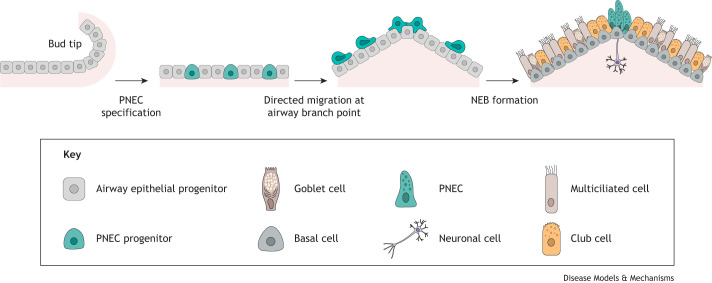
**Schematic of NEB formation during airway development.** NEB formation follows a series of steps. (1) Pulmonary neuroendocrine cell (PNEC) specification: PNECs are the first epithelial cell type to differentiate in the developing airway and appear scattered throughout the epithelium. (2) Directed migration at airway branchpoints: PNECs detach from neighbouring cells and migrate to airway branch points where they aggregate in clusters. (3) Neuroepithelial body (NEB) formation: PNEC clusters are innervated by nerve fibres and the NEB microenvironment is established.

After their specification during lung development, PNECs are found either as scattered solitary cells or enriched in small clusters, referred to as neuroepithelial bodies (NEBs). NEBs are highly innervated and are often found at the branching points of airway tubules (Kuo and Krasnow, 2015; Lauweryns and Peuskens, 1972; Stahlman et al., 1987) (Fig. 1B). Live imaging and PNEC lineage-tracing studies revealed that during embryonic mouse lung development, NEBs are formed through the migration of individual PNECs to airway branch points, where they cluster together (Kuo and Krasnow, 2015; Noguchi et al., 2015) (Fig. 3). After cluster formation, afferent and efferent nerve fibres extend and ramify on the NEBs (Lauweryns and Van Lommel, 1987). In mice, NEBs, rather than solitary PNECs, are selectively supplied by nerves, whereas in humans, there is evidence that solitary PNECs are also innervated (Guha et al., 2012). The innervation of NEBs in this way and their enrichment at bifurcation points where air is trapped suggest that PNECs fulfil a key function as sensors of various stimuli in the lung (Xu et al., 2020).

The function of PNECs as sensors was first described five decades ago. In response to airway hypoxia, increased exocytosis of dense-core vesicles was observed in rabbit lungs (Lauweryns et al., 1977, 1978). Later, the detection of an oxygen-binding protein on the PNEC cell membrane provided further support for the idea that PNECs might function as oxygen sensors in the airway (Youngson et al., 1993). Chemical stimuli have also been shown to trigger responses in PNECs. For example, normal fetal hamster PNECs respond to nicotine by increasing their secretion of 5-HT (Plummer et al., 2000). Human PNECs found in cultures of tracheobronchial epithelium have been shown to express olfactory receptors and to release 5-HT and CGRP in response to volatile chemicals (Gu et al., 2014). In addition to these chemical signals, PNECs can sense mechanical stress. Mechanical stretching of PNECs isolated from rabbit fetal lungs led to the increased release of 5-HT (Pan et al., 2006b). Thus, PNECs can sense different environmental stimuli and respond to these through the release of bioactive compounds.

The bioactive compounds secreted by PNECs influence lung physiology. PNEC signals can, for example, regulate immune system responses. In a mouse model of congenital diaphragmatic hernia with genetic ablation of *Robo1* and *Robo2*, PNEC product secretion was increased, which led to increased infiltration of macrophages and other immune cells into the lung (Branchfield et al., 2016). In a mouse model of allergic asthma, Sui et al. (2018) demonstrated that PNEC-secreted bioactive molecules amplify allergic asthma responses by triggering immune reactions and increasing mucous secretion in the airway. Specifically, PNEC-derived CGRP recruits group 2 innate lymphoid cells to the lung and stimulates their secretion of cytokines. In addition, the neurotransmitter γ-aminobutyric acid (GABA), also secreted by PNECs, promotes goblet cell hyperplasia, leading to increased mucous secretion (Sui et al., 2018).

In addition to immune cells and goblet cells, other cells have been implicated as targets of PNEC signals. Receptors for signalling molecules secreted by PNECs are expressed by a wide array of cell types, including airway epithelial cells, immune cells, neurons, endothelial cells and airway smooth muscle cells, indicating that multiple cell types respond to PNEC-derived signals (Kuo et al., 2022). Myoepithelial cells (Box 1) wrapped around the airway submucosal glands (Box 1) have been recently reported to respond to ATP released from PNECs, triggering myoepithelial contraction and, in turn, promoting mucous ejection (Yu et al., 2022b). Basal cells also respond to CGRP. In the context of hypoxic injury in the mouse airway epithelium, CGRP promoted basal cell proliferation and differentiation into PNECs. Intranasal administration of CGRP was sufficient to mitigate hypoxia-induced airway injury (Shivaraju et al., 2021). This suggests that, in response to lung injury, PNECs regulate protective tissue responses.

Indeed, the NEB microenvironment retains a source of facultative epithelial stem cells that mediate airway repair in response to injury (Hong et al., 2001; Reynolds et al., 2000). Two epithelial stem cell populations have been detected in murine NEBs. One population, called variant club cells (vCCs; Box 1), are resistant to naphthalene injury (Box 1) and selectively express uroplakin-3a (*Upk3a*) (Guha et al., 2012, 2017). The second population constitutes a subset of murine PNECs with stem cell features (NE^stem^ cells; Box 1), which selectively proliferate and transdifferentiate to other pulmonary fates following naphthalene injury *in vivo* (Ouadah et al., 2019). Therefore, PNECs may reflect a heterogenous rather than a homogenous population of cells that harbour different characteristics and perform different functions. Consistent with this, two research groups, through integrated scRNA-seq and spatially resolved transcriptomics of the human embryonic fetal lung, independently characterised distinct groups of PNECs present during early lung development. These groups of PNECs were distributed differently along the airway and were defined as progenitor PNECs, gastrin-releasing peptide (GRP)-positive PNECs and ghrelin (GHRL)-positive PNECs (Sountoulidis et al., 2023; He et al., 2022). In the adult lung, fully differentiated PNECs also display a substantial level of heterogeneity. scRNA-seq analysis of adult mouse and human airways identified an extensive repertoire of neuropeptide and peptide hormone (‘peptidergic’) genes expressed in PNECs, revealing an extraordinary diversity and myriad combinations of such peptidergic genes expressed across PNECs (Kuo et al., 2022). Given the known link between PNECs and cancer, it is tempting to speculate that different subtypes of pulmonary neuroendocrine cancers originate from different PNEC subsets. To address this question, studies focusing on how PNEC neoplasms arise, evolve into specific molecular subgroups and then progress are needed.

Progress in PNEC research has drawn attention to this previously underappreciated, rare cell population of the lung, which is emerging as a relevant modulator of lung physiology. The association of PNECs with lung pathologies, such as chronic respiratory diseases and pulmonary neuroendocrine cancers, indicates that research investigating the pathophysiological role of PNECs could be of therapeutic value for treating lung diseases. In the rest of this Review, we summarise what is known about the association of PNECs with lung pathologies, focusing on how new experimental disease models, such as organoids derived from either human pluripotent stem cells (hPSCs) or tissue stem cells, can help us to better understand the contribution of PNECs to respiratory diseases.

## How do PNECs play a role in respiratory diseases?

The airway epithelium is constantly exposed to external air and is thus liable to injury from pollutants and pathogens carried by aerosols. The lung has therefore evolved several defence responses to deal with diverse types of injury (Planer and Morrisey, 2023). Chemical insults and viral infection can both lead to an altered cellular milieu and, ultimately, to the remodelling of the airway. The hyperactivation of PNECs resulting in increased secretion of bioactive compounds and/or PNEC hyperplasia has been detected in response to an array of insults, including hypoxia, hyperoxia (Box 1) and smoking (Aguayo et al., 1989; Ratcliffe et al., 2016; Shenberger et al., 1997). In some cases, PNECs appear to play a protective role in the airway epithelium. For instance, as we discussed previously, in response to airway hypoxia, PNECs promote basal cell proliferation, thereby mitigating hypoxia-induced injury (Shivaraju et al., 2021). Likewise, exposure to hyperoxia, which increases airway smooth muscle wall thickness and heightens airway reactivity, is also associated with PNEC hyperplasia (Shenberger et al., 1997). This increase in PNEC numbers, coupled with the accompanying increased neuropeptide production in response to hyperoxia, is thought to be protective and counteract airway hyperresponsiveness. These effects are attributed specifically to PNEC-secreted CGRP, which relaxes tracheal smooth muscle (Bhogal et al., 1994).

In contrast, the presence of too many PNECs can also be damaging. Increased PNEC numbers and/or products have been reported in lung diseases, such as CF, asthma and COPD (Johnson et al., 1988; Xu et al., 1999). Hyperoxia-induced PNEC hyperplasia is thought to mediate lung injury in patients with bronchopulmonary dysplasia, a chronic lung disease associated with oxygen supplementation of premature infants. In a baboon model of bronchopulmonary dysplasia, some of the lung defects associated with the disease could be prevented by treatment of the animals with a GRP-blocking antibody, directly linking PNEC-secreted GRP to the disease phenotype (Sunday et al., 1998). PNECs are also strongly associated with lung neuroendocrine cancers and this association could be linked to the dysregulation of the protective and regenerative functions of this cell population. A better understanding of the role that PNECs play in respiratory diseases and lung cancers could contribute to the development of novel therapeutic strategies that target the neuroendocrine system in the lung. In this section, we review current evidence concerning the contribution of PNECs to respiratory diseases.

### CF

CF is caused by mutations in the CF transmembrane conductance regulator (*CFTR*) gene (Riordan et al., 1989). *CFTR* is expressed by multiple epithelial cell types and encodes a chloride channel (Plasschaert et al., 2018; Shah et al., 2022). In the human airway, impaired CFTR function results in impaired mucous clearance and host defence. As a result, patients with CF suffer persistent bacterial infections and respiratory insufficiency (Elborn, 2016). It is unclear whether CFTR dysfunction influences PNECs in a cell-intrinsic manner. Other airway epithelial cells, including the recently discovered ionocytes, express *CFTR* at levels much higher than those observed in PNECs (Okuda et al., 2021). Nevertheless, multiple lines of evidence suggest that PNECs contribute to CF disease pathology.

**Fig. 4.**
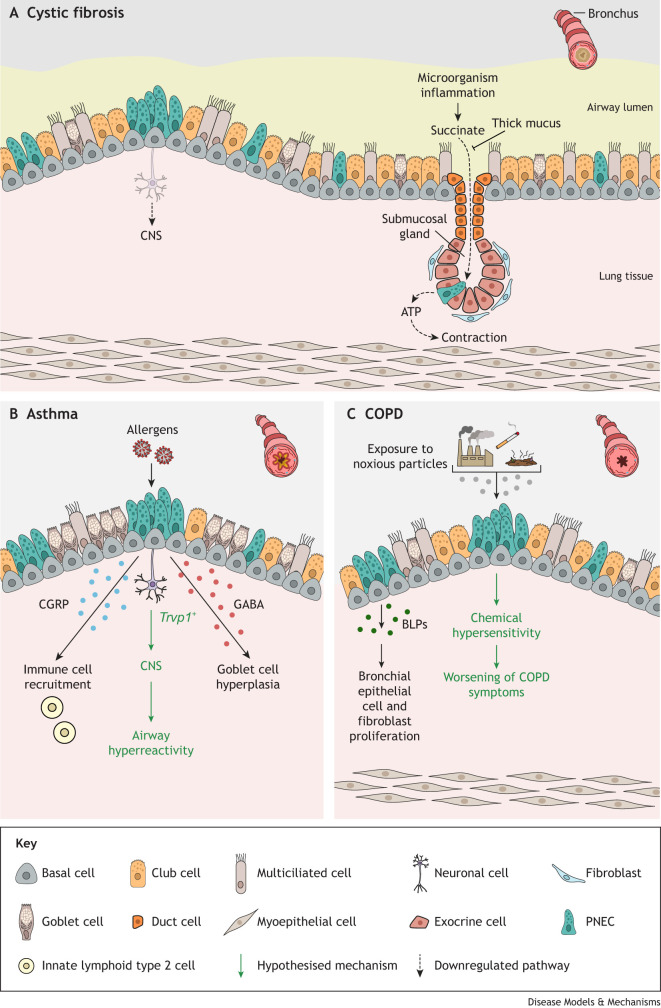
**PNEC involvement in three respiratory diseases.** In panels A-C, the existing evidence for the role of pulmonary neuroendocrine cells (PNECs) in each respiratory disease is shown. Additional hypothesised mechanisms are highlighted with green text and arrows. Each schematic also shows a cross section of a bronchus in the described disease (top right), showing mucous accumulation in cystic fibrosis, inflammation in asthma and fibrosis in chronic obstructive pulmonary disease (COPD). (A) In cystic fibrosis, neuroepithelial bodies (NEBs) have a reduced number of nerve connections, potentially leading to impaired communication between PNECs and the central nervous system (CNS). PNEC sensing of the inflammatory molecule succinate is hindered by the persistent mucus obstruction of cystic fibrotic airways. Consequently, PNECs cannot react to succinate and release adenosine triphosphate (ATP), which stimulates the contraction of myoepithelial cells and glandular mucous ejection, thus promoting respiratory defences. (B) PNECs secrete γ-aminobutyric acid (GABA), which promotes goblet cell hyperplasia, as well as calcitonin gene-related peptide (CGRP), which enhances immune responses and airway inflammation. Increased PNEC numbers in asthmatic lungs might thus disrupt immune responses, contributing to the pathogenesis of asthma. Neurons expressing *Trpv1*, implicated in the development of allergen-induced airway hyperreactivity in asthma, can contact the basal side of PNECs. An additional hypothesised mechanism is that PNECs might thus interact with *Trpv1*^+^ neurons, and PNEC hyperplasia might worsen airway hyperreactivity in asthma by enhancing the activation of *Trpv1*^+^ neurons. (C) Bombesin-like peptides (BLPs) released by PNECs are mitogenic for human bronchial epithelial cells and lung fibroblasts. Consequently, PNEC hyperplasia induced by smoking and the increased production and secretion of PNEC products might contribute to airway thickening and to peribronchiolar fibrosis associated with COPD. An additional hypothesised mechanism is that PNEC hyperplasia might amplify PNEC chemosensory function, causing heightened airway responsiveness to chemical stimuli and leading to heightened responses to inhaled chemical stimuli. This could potentially trigger airway inflammation and exacerbate episodes characterised by worsening symptoms.

As is the case for all the respiratory diseases described in this Review, PNEC numbers and their secreted products are increased in the lungs of patients with CF (Johnson et al., 1988). PNEC hyperplasia in CF is presumed to be a response to the chronic hypoxia experienced by these patients. The consequences of increased PNEC numbers in the context of the altered cellular milieu and chronic inflammation present in CF lungs are likely to contribute to the pathology of this disease. A potential problem in the lungs of patients with CF is that persistent mucus obstruction prevents signalling molecules from diffusing properly across the epithelium, thereby preventing PNECs from receiving or responding to these signals (Yu et al., 2022a). PNEC sensing of the inflammatory molecule succinate is compromised in the lungs of pigs with CF (Yu et al., 2022a). PNECs in the submucosal glands respond to succinate by releasing ATP, which stimulates contraction in neighbouring myoepithelial cells, eliciting glandular mucous ejection (Yu et al., 2022a). This mechanism is required to promote host respiratory defences (Yu et al., 2022a). Given that PNEC chemosensory functions are thought to be protective, impaired chemosensing by PNECs in CF could impair host respiratory defence and repair. Indeed, attenuated PNEC communication might be a general theme in the lungs of patients with CF. It has been observed that NEBs of *Cftr* knockout mice show reduced levels of innervation compared to NEBs from wild-type mice (Pan et al., 2006a). Fewer nerve connections in NEBs could result in blunted PNEC communication with the nervous system. These observations and experimental results in animal models support the hypothesis that PNECs contribute to CF pathogenesis (Fig. 4A). Nevertheless, several questions remain. Is PNEC hyperplasia in CF a response to hypoxic conditions or a mechanism to counterbalance the reduced PNEC chemosensation caused by mucous obstruction? Could CFTR be involved in PNEC development or neuropeptide secretion, and, if so, could this explain the reduced NEB innervation reported in mice lacking CFTR? Further studies are required to address these questions.

### Asthma

Asthma is a chronic disease of the conducting airways that affects over 300 million people worldwide (Vos et al., 2012). It is characterised by inflammation and tissue remodelling as a result of airway hyperreactivity (Papi et al., 2018). Traditionally, two forms of asthma have been defined in the clinic: allergic asthma, resulting from sensitisation to common allergens, involves eosinophilic airway inflammation and hyperreactivity, driven by adaptive T helper 2 cells, and non-allergic (intrinsic) asthma operates independently of the adaptive immunity, with group 2 innate lymphoid cells governing airway inflammation and hyperreactivity (Lambrecht and Hammad, 2015). Cells of the immune system are considered to be the main drivers of disease symptoms (Holgate, 2012); however, this does not exclude a role for the lung epithelium. An altered airway epithelium characterised by PNEC hyperplasia is observed in the lungs of patients with non-allergic asthma (Stanislawski et al., 1981). This increase in PNECs in the lungs of patients with asthma is thought to alter PNEC–immune cell interactions, promoting aberrant immune responses and directly contributing to the disease pathology. Sui et al. (2018) demonstrated that PNECs are essential for allergen-induced asthma-like responses in mice. *Ascl1* knockout mice, which lack PNECs, showed a blunted mucosal type 2 response (Box 1) after ovalbumin allergen challenge, which could be rescued by the intratracheal administration of CGRP and GABA (Branchfield et al., 2016). In addition, Branchfield et al. (2016) showed that upregulation of PNEC products in *Robo1* and *Robo2* double-mutant mice, a genetic model of congenital diaphragmatic hernia, increased immune responses and airway inflammation. Therefore, increased PNEC number in asthmatic lungs can dysregulate immune responses, contributing to asthma pathogenesis.

Given that PNECs are known to contact neurons and are assumed to interact with them, PNEC hyperplasia could also affect PNEC interaction with the nervous system, a known mediator of airway hyperreactivity in asthma. In a preprint, Su et al. (2023 preprint) mapped the neural circuitry involved in establishing allergen-induced airway hyperreactivity in a mouse model of asthma. Interestingly, they showed that *Trpv1*^+^ neurons, the ablation of which led to reduced airway hyperreactivity in allergen-challenged mice (Tränkner et al., 2014), expressed multiple receptors for PNEC-secreted ligands (Su et al., 2023 preprint). Moreover, *Trpv1*^+^ neurons have been shown to contact the basal side of individual PNECs (Su et al., 2022). Thus, PNECs may interact with *Trpv1*^+^ neurons, and PNEC hyperplasia might exacerbate airway hyperreactivity in asthma. Together, these studies suggest that PNECs contribute to asthma pathology (Fig. 4B). Further studies are required to better address whether PNECs are directly involved in establishing airway hyperreactivity and to reveal the detailed mechanisms by which PNECs are activated after an allergen challenge.

### COPD

COPD is a common respiratory disease and the third leading cause of death worldwide (Vos et al., 2020). It is characterised by the chronic obstruction of the small airways (bronchiolitis) and by alveolar damage (emphysema), which leads to shortness of breath in response to physical exertion (dyspnea) and to a persistent cough (Barnes et al., 2015). The inhalation of cigarette smoke or of other harmful particles is considered a primary driver of COPD, through persistent pulmonary injury that leads to chronic inflammation and tissue remodelling (Mathioudakis et al., 2020). Cigarette smoke has been shown to shift the composition of the airway epithelium – a common feature of most chronic lung diseases – by increasing PNEC numbers (Aguayo et al., 1989; Goldfarbmuren et al., 2020; Gu et al., 2014).

Even though a clear understanding of whether PNEC hyperplasia in smokers' lungs can promote COPD is still lacking, several mechanisms have been proposed. Bombesin-like peptides (BLPs) secreted by PNECs are mitogenic for human bronchial epithelial cells and lung fibroblasts (Aguayo et al., 1990; Willey et al., 1984). As such, they might play a role in the process of airway narrowing, driving airway thickening and peribronchiolar fibrosis in COPD. Supporting this hypothesis, PNEC hyperplasia and increased BLPs in patients who have never smoked appear to cause COPD-associated symptoms of peribronchiolar fibrosis and chronic airflow obstruction (Aguayo et al., 1992). Thus, PNEC hyperplasia might contribute to the development of COPD by promoting aberrant airway remodelling. Further supporting PNEC hyperplasia as a driver of COPD pathogenesis, an increase in PNECs is thought to amplify their chemosensory function, causing heightened airway responsiveness to chemical stimuli. In primary human cell cultures, volatile chemicals have been shown to activate PNECs that express olfactory receptors (Gu et al., 2014). It is tempting to speculate that PNEC hyperplasia in patients with COPD might cause exaggerated reactions to inhaled chemical stimuli that promote airway inflammation, resulting in episodes (called exacerbations) in which symptoms worsen. COPD exacerbations are associated with poor prognosis and have been linked to exposure to air pollutants (Barnes et al., 2015). These observations suggest that PNEC hyperplasia contribute to the development of COPD (Fig. 4C). Studies that address how PNECs respond to air pollutants associated with COPD development and exacerbations are key to understanding how PNECs might contribute to COPD pathogenesis. Future work will also need to investigate whether PNECs are the major contributors that drive airway thickening and airflow obstruction in COPD.

### Diffuse idiopathic PNEC hyperplasia

Diffuse idiopathic PNEC hyperplasia (DIPNECH) is a rare pulmonary disorder, first described and defined by Aguayo et al. (1992). Patients with DIPNECH manifest symptoms, including cough, dyspnea, wheezing, and mixed obstructive and restrictive defects in pulmonary function tests. DIPNECH is recognised by the World Health Organization as a preinvasive, possibly pre-neoplastic condition (Travis et al., 2015). In most patients, DIPNECH manifests as an indolent and nonprogressive disorder, although a few patients can progress to severe airflow obstruction or develop metastatic carcinoid tumours (Davies et al., 2007; Flint et al., 2019). The symptoms of DIPNECH are accompanied by primary PNEC proliferation that is detectable on histological analyses. Such neuroendocrine cell proliferations include an increase in scattered single neuroendocrine cells, the presence of small nodules (NEBs) or a linear proliferation of PNECs (Aguayo et al., 1992). When neuroendocrine cell proliferations in DIPNECH extend beyond the basement membrane, they are defined as either tumourlets (<5 mm; Box 1) or carcinoid tumours (>5 mm).

PNEC hyperplasia can also be found in other settings, including in smoking-related diseases, reactive proliferation in primary or metastatic cancers, or in response to infection (Rossi et al., 2016). However, PNEC hyperplasia associated with the clinical symptoms of DIPNECH is considered to be a distinct pathological entity. The most effective treatment for patients with DIPNECH is somatostatin analogues (SSAs), which can be effective in palliating chronic respiratory symptoms (Al-Toubah et al., 2020). It is well known that SSAs prevent gastrointestinal neuroendocrine tumours from secreting bioactive substances (Grozinsky-Glasberg et al., 2008). The fact that SSAs effectively treat cough and dyspnea in patients with DIPNECH suggests that the production of bioactive molecules by PNECs in DIPNECH is what causes these symptoms (Al-Toubah et al., 2020). Although evidence clearly linking DIPNECH with carcinogenesis is still lacking, carcinoid tumours in a patient with proven DIPNECH is a predictor of poorer prognosis. In a cohort of patients with resected carcinoid tumours, the occurrence of a more aggressive type of carcinoid (atypical carcinoid), as well as the rate of mediastinal lymph node invasion, were both significantly higher in patients with DIPNECH than in those without DIPNECH (Prieto et al., 2021). Thus, carcinoids in patients with DIPNECH might represent a separate entity from carcinoid tumours present in those without DIPNECH. It follows then that carcinoids that arise from DIPNECH lesions might represent tumours initiating from a different PNEC than carcinoids arising *de novo* in the absence of prior DIPNECH. Studies are required to test this hypothesis and to elucidate the underlying mechanisms to explain the higher prevalence of atypical carcinoids in patients with DIPNECH. Future work is also needed to understand what triggers PNEC hyperplasia in patients with DIPNECH and how PNEC proliferations contribute to the development of the pathology of this disease.

### Lung cancer

**Fig. 5.**
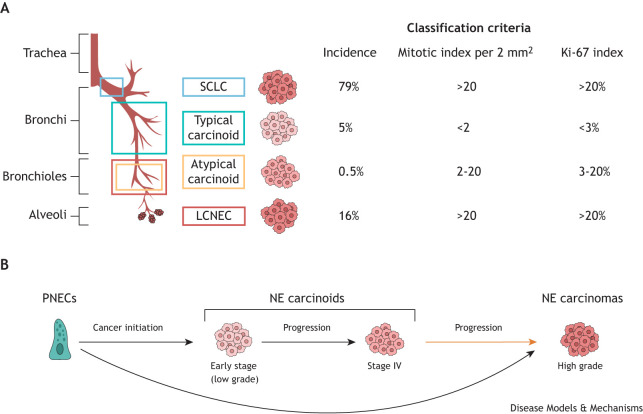
**Lung neuroendocrine cancer classification and progression.** (A) The typical locations throughout the human airway in which neuroendocrine (NE) cancer subtypes, namely, small-cell lung cancer (SCLCs, blue), typical carcinoids (green), atypical carcinoids (yellow) and large-cell NE carcinomas (LCNECs, red), are shown. The incidence, as a percentage of lung neuroendocrine cancers (Rindi et al., 2018) and histological features used to classify these subtypes are also shown. Mitotic index is defined as the ratio of the number of cancer cells undergoing mitosis to the total number of cancer cells, and the Ki-67 index indicates the speed of cancer cell proliferation. (B) Model illustrating lung NE cancer initiation and progression, in which lung NE cancers mainly initiate from pulmonary NE cells (PNECs, black arrows), and in which some carcinoids (yellow arrow) can progress to becoming tumours resembling high-grade carcinomas.

One-third of all human lung cancers exhibit signs of neuroendocrine differentiation (Travis, 2010). Lung neuroendocrine neoplasms (NENs) have a wide spectrum of clinical behaviours. They can be divided into four histological variants: SCLCs, LCNECs, atypical carcinoids and typical carcinoids (Fig. 5A) (Rekhtman, 2022). Clinically, SCLCs and LCNECs are high-grade carcinomas (Box 1) with a 5-year survival rate of 5% and 15-25%, respectively (Govindan et al., 2006; Travis et al., 2015). By definition, neuroendocrine carcinomas have a mitotic index (Box 1) of more than 20 (per 2 mm^2^). The overall 10-year survival for atypical carcinoids, classified as intermediate-grade tumors, ranges from 91% for stage I tumours to 18% for stage IV tumours, whereas for typical carcinoids, classified as low-grade tumors, the 10-year survival ranges from 98% for stage I tumours to 49% for stage IV tumours (Yoon et al., 2019). The mitotic index for carcinoids ranges from 2 to 20 (per 2 mm^2^).

SCLC is the most common and aggressive form of lung NEN, characterised by near-ubiquitous loss of function of the *RB1* and *TP53* tumour suppressor genes (George et al., 2015). PNECs are recognised as a cell of origin of SCLC. Mouse studies have demonstrated that inactivation of *Rb1* and *Tp53* in *Cgrp*-expressing PNECs leads to SCLC (Sutherland et al., 2011; Song et al., 2012). Moreover, NE^stem^ cells were shown to proliferate slowly and continuously right after *Rb1* and *Tp53* deletion in *Ascl1*-expressing mouse PNECs, suggesting that this subpopulation of PNECs represents tumour-initiating cells for SCLC (Ouadah et al., 2019). Nevertheless, NE^stem^ cells are unlikely to be the exclusive cell of origin for SCLC; other cells are also considered to give rise to SCLC (Huang et al., 2018; Yang et al., 2018b; Ferone et al., 2020). Highlighting the heterogeneity of SCLC, genomic and transcriptomic profiling of mouse and human SCLC has revealed that SCLC comprises four distinct molecular subtypes (SCLC-A, SCLC-N, SCLC-P and SCLC-Y) characterised by the predominant expression of the ASCL1, NEUROD1, POU2F3 or YAP1 transcription factors (Ireland et al., 2020). Each SCLC subtype is suggested to be determined by the identity of the tumour-initiating cell, as well as by genetic drivers (Yang et al., 2018b). For example, amplification or overexpression of the gene *MYC* in SCLC is postulated to drive the evolution of SCLC-A into SCLC-N and then to SCLC-Y. Time-course scRNA-seq analysis of ASCL1^+^ SCLC cultured *in vitro* demonstrated the ability of *MYC* to promote SCLC subtype evolution, indicating that the SCLC-A, SCLC-N and SCLC-Y subtypes might represent different stages of SCLC progression (Ireland et al., 2020). The SCLC-P subtype has been proposed to arise from another rare chemosensory lung cell type, the tuft cell, which shares the expression of several markers with SCLC-P (Huang et al., 2018). Accordingly, SCLCs in genetically engineered mouse models could not give rise to the POU2F3^+^ SCLC-P variant when the tumour was initiated in either PNECs, club cells or alveolar cells using cell type-specific promoters. SCLC-P only arose in this model when tumours were initiated with a general promoter (Ireland et al., 2020). Further highlighting how the cell of origin can play a role in the evolution of SCLC tumours, Yang et al. (2018b) demonstrated that the same genomic alteration in different cell types drives different gene expression programs that enable metastatic progression. Together, these studies have led to the identification of different therapeutic sensitivities across specific SCLC subtypes (Cardnell et al., 2017; Huang et al., 2018; Mollaoglu et al., 2017; Saunders et al., 2015). However, adopting subtype-specific therapeutic strategies might not represent the most effective approach to treating SCLC, considering the dynamic evolution of this cancer and that individual SCLC tumours comprise cells belonging to different subtypes (Huang et al., 2018; Stewart et al., 2020). Future studies that investigate how SCLC progresses and gives rise to intratumoural heterogeneity should inform the development of new therapeutic strategies.

As with SCLCs, LCNECs and carcinoids are also thought to originate from PNECs (Fisseler-Eckhoff and Demes, 2012). However, less is known about these other histological tumour subtypes. An analysis of mutational and transcriptional patterns in LCNECs found that LCNEC comprises two transcriptionally dissimilar genomic subgroups: type I LCNECs with bi-allelic *TP53* along with *STK11* and/or *KEAP1* alterations, which exhibit a neuroendocrine expression profile, and type II LCNECs, which are enriched for bi-allelic inactivation of *TP53* and *RB1* and are characterised by the reduced expression of neuroendocrine markers and the upregulation of immune-related pathways (George et al., 2018). Although deleterious mutations in the two tumour suppressors genes *TP53* and *RB1* are frequently associated with carcinomas, these are rare events within carcinoids, which have a lower mutational burden compared to that of carcinomas, and a higher prevalence of mutations in genes involved in chromatin remodelling (Fernandez-Cuesta et al., 2014). A subgroup of carcinoids, termed ‘supra-carcinoids’, has been identified, which has a carcinoid morphology but molecular characteristics similar to those of LCNEC (Alcala et al., 2019). In this first study, supra-carcinoids accounted for 5.5% of the pulmonary carcinoids analysed (Alcala et al., 2019). Further studies are needed to determine the exact frequency of supra-carcinoids. These observations relating to supra-carcinoids support the hypothesis that, in some rare cases, pulmonary carcinoids can progress into tumours that resemble high-grade malignant carcinomas. The apparently low frequency of this progression event suggests that supra-carcinoids arise from a different cell of origin than the other more common subtypes of carcinoids. More research will be required to explore the clinical implications and specific molecular processes underpinning how PNEC neoplasms arise, evolve into specific molecular subgroups and progress from low- or intermediate- grade tumours into high-grade malignant carcinomas (Fig. 5B). We anticipate that future advancements in *in vitro* models of the human airway epithelium will prove valuable for investigating the pathological significance of PNECs in respiratory diseases.

## Model systems for studying the human airway epithelium and PNECs

The progress of research into PNECs has been significantly hindered by their rarity, the absence of established isolation protocols and the challenges associated with the long-term cultivation of human cells. These challenges are beginning to be addressed through the creation of *in vitro* models of the human airway, consisting of pseudo-stratified epithelia that contain different lung cell types and recapitulate many features of the *in vivo* airway epithelium. Cells cultured in these systems can be manipulated to differentiate into PNECs, facilitating the study of these rare cells. Here, we describe several *in vitro* models of the human airway epithelium (Table 1) that could be used to explore the pathological role of PNECs in respiratory diseases and that might guide the development of new therapies to target the pulmonary neuroendocrine system.

**Table 1.**
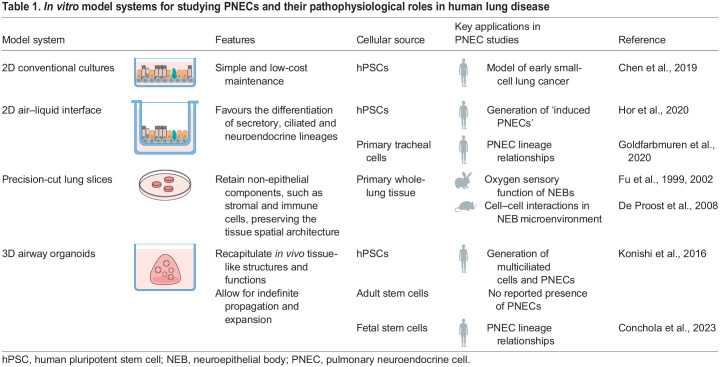
*In vitro* model systems for studying PNECs and their pathophysiological roles in human lung disease

### Air–liquid interface cultures

Air–liquid interface (ALI) cultures are the most commonly used models to study the airway epithelium and aim to recapitulate the microenvironment of airway epithelial cells (Whitcutt et al., 1988; Baldassi et al., 2021). In the lung, epithelial cells make up the luminal surface of the respiratory tract and create a tight barrier at the interface between the air in the airways and an intricate capillary network found on the basal side of the airways. ALI systems recreate an air interface and a blood flow-like interface. In ALI culture, cells grown on a porous membrane are exposed to air on their apical surface and are nourished through their basolateral surface, which is in contact with a liquid medium. This configuration allows ciliated and secretory cells to differentiate (Ross et al., 2007; Yamaya et al., 1992), indicating that the exposure of these airway epithelial cells to air promotes their maturation. PNECs have been generated from human induced pluripotent stem cells (hiPSCs) in ALI conditions (Hor et al., 2020). These so-called ‘induced PNECs' could provide researchers with an inexhaustible resource with which to investigate PNEC pathophysiology in lung diseases. These cells are transcriptomically similar to human fetal lung PNECs (Hor et al., 2020). However, their similarity to PNECs in the adult human lung remains unclear, leaving open the question as to their potential application to model pathological conditions of adult respiratory diseases. The generation of PNECs from primary human tracheal cells grown in ALI systems has also been reported (Mou et al., 2021). Although the authors of this study did not address whether these derived PNECs resemble adult human PNECs, it is likely that PNECs derived from primary airway tissue will more accurately resemble their *in vivo* mature counterparts. Using these tracheal-derived primary cell ALI cultures in combination with CRISPR knockout studies, Goldfarbmuren et al. (2020) investigated lineage relationships among rare airway epithelial cell types: PNECs, tuft cells and ionocytes. Their findings point to a branched lineage progression, in which tuft-like cells give rise to both ionocytes and PNECs. However, lineage-tracing studies are needed to confirm this lineage relationship between tuft cells and PNECs. In a preprint, Waghray et al. (2023 preprint) described an equivalent progenitor population found in the large airways of human adults through deep scRNA-seq (Waghray et al., 2023 preprint). Leveraging human ALI cultures, this replicative rare cell progenitor population was shown to have the potential to differentiate into either mature tuft cells or ionocytes and was therefore termed tuft-ionocyte progenitor (TIP) cell. The authors did not find any evidence that TIP cells could give rise to PNECs. Intriguingly, they showed that cytokines associated with asthma and CF altered the differentiation of TIP cells (Waghray et al., 2023 preprint). Perhaps in the context of other human respiratory diseases, TIP cells could be pathologically redirected to produce PNECs.

ALI culture systems provide a potentially useful *in vitro* tool with which to study respiratory airways and PNEC biology in healthy and diseased states (Baldassi et al., 2021). However, it is important to note that these systems often require extended differentiation periods before PNECs emerge. Furthermore, the long-term expansion potential of primary or hiPSC-derived PNECs in these systems has yet to be fully determined. To overcome these limitations, further research is needed to optimise the culture techniques used to derive PNECs in ALI culture systems.

### Precision-cut lung slices

Precision-cut lung slices (PCLSs) offer promise as an *ex vivo* platform for translational research. PCLSs are obtained by infusing freshly retrieved whole lungs or individual lobes with warm low-melting agarose solution, which fills the airway to maintain the expanded state of the lung and prevents tissue damage during the slicing process (Placke, 1987). The solidified infused tissue is then sliced with a vibrating microtome, resulting in slices of uniform thickness (100-500 μm), which can be preserved and studied *ex vivo* (Liu et al., 2019).

A key advantage of PCLSs is that they retain all cell types present in the original lung tissue, including crucial non-epithelial components, such as stromal cells and immune cells. This provides researchers with the opportunity to explore interactions between the immune system and other resident lung cells (Temann et al., 2017). For instance, PCLSs could be used to investigate how the interactions between PNECs and immune cells influence immune responses and contribute to respiratory diseases, such as asthma and COPD. PCLSs also preserve the spatial cellular architecture of lung tissue, which enables ‘intact’ NEBs from rabbits and mice to be studied in a relatively native environment. Fu et al. (1999, 2002) used PCLSs from rabbit and hamster lungs to study the effect of hypoxia on NEBs. These two studies revealed that NEBs release 5-HT under hypoxic conditions and in response to ATP, and that this response was mediated through the inhibition of voltage-gated K^+^ channels present on NEB cells (Fu et al., 1999, 2002). In another study, De Proost et al. (2008) performed live-cell imaging of NEBs in murine PCLSs to investigate the response of NEBs and surrounding cells to applied stimuli. In response to a high K^+^ concentration, NEB cells and vCCs that encircle them increased their cytoplasmic Ca^2+^, whereas other epithelial cells did not. Remarkably, vCCs exhibited a delayed response in comparison to NEB cells, hinting at an indirect activation mechanism mediated by NEBs (De Proost et al., 2008). Together, these studies highlight the benefit of using PCLS approaches to conduct comparative studies of NEBs, at different developmental stages and in different species, including humans, for which NEB research is limited.

PCLSs have also been used to investigate human respiratory diseases (Liu et al., 2019). However, PCLSs undergo a gradual decline in cellular integrity and functionality over time. PCLSs that have been cultured for a prolonged period reduce their production of cytokines and chemokines when exposed to external stimuli (Liu et al., 2019; Temann et al., 2017). Moreover, after the first week of culture, the airway epithelium in PCLSs undergoes a significant decline in cilia functionality, whereas the alveolar epithelium and vascular system undergo severe cell loss within the first 2 weeks (Preuß et al., 2022). The short window of cellular viability in PCLSs thus prevents the modelling of the long-term effects of respiratory diseases, such as chronic inflammation and cancer progression, and also implies that gene-editing experiments would be nearly impossible or extremely challenging to conduct in this system. The viability of PNECs and their functionality over time in human PCLSs is unclear and future studies will be needed to address this. This will lay the foundation for utilizing human PCLSs as a valuable tool in which to investigate the biology of PNECs and their impact on respiratory diseases.

### hPSC-based cultures

hPSCs – either embryonic stem cells or hiPSCs – provide an unlimited source of cells that can be differentiated into lung progenitor cells. The ability to generate airway epithelia from hPSCs has provided an effective approach to study lung development, model respiratory diseases and conduct drug screening (Goldsteen et al., 2021). Several differentiation protocols to derive airway epithelial cells from hPSCs in two-dimensional (2D) cultures (Firth et al., 2014; Huang et al., 2014; Mou et al., 2012; Wong et al., 2012) and in three-dimensional (3D) organoids (Chen et al., 2017; Dye et al., 2015; Gotoh et al., 2014; Konishi et al., 2016; McCauley et al., 2017) have been established that aim to recapitulate lung development *in vitro* through the precise activation and/or inhibition of key signalling pathways in a carefully timed manner.

Airway epithelial lineages arise from progenitors present on growing bud tips during branching morphogenesis (Rawlins et al., 2009), which in turn emerge from the anterior ventral side of the definitive endoderm (Morrisey and Hogan, 2010) (Fig. 2). Therefore, the process of differentiating hPSCs into pulmonary tissue commences with differentiation into the definitive endoderm, then the anterior foregut endoderm (AFE), the ventral AFE and, finally, the specification of lung and airway lineages. The activation of activin A signalling drives the differentiation of hPSCs into the definitive endoderm, which can be further anteriorised and differentiated towards the AFE through the dual inhibition of tansforming growth factor β (TGF-β) and bone morphogenetic protein (BMP) signalling (Green et al., 2011). The subsequent addition of different combinations of Wingless-related integration site (WNT) signals, fibroblast growth factors (FGFs), BMPs, sonic hedgehog protein (SHH), keratinocyte growth factors (KGFs) and retinoic acid can result in the formation of lung progenitors and cell cultures that express a wide range of lung epithelial markers (Firth et al., 2014; Huang et al., 2014; Mou et al., 2012; Wong et al., 2012). Chen et al. (2017) reported the creation of 3D hPSC-derived lung organoids that contained mesoderm and developed into branching structures, resembling the proximodistal architecture and cell lineages of the airway tree. Interestingly, when transplanted under the kidney capsule of immunodeficient mice, these organoids were also able to grow NEBs. Whether these organoids contained significant numbers of PNECs when they were grown *in vitro* was not reported. It would be of interest to conduct future experiments to investigate whether manipulating Notch signalling, which is known to influence proximal and distal cell fate differentiation (Yamamoto et al., 2017), can promote neuroendocrine fate in these organoids.

Some efforts have been made to specifically differentiate PNECs from 2D and 3D hPSC-derived cultures. As discussed in Box 2, Notch signalling inhibition drives the neuroendocrine fate. Chen et al. (2019) used this knowledge to induce PNECs from hPSC-derived lung progenitors grown in two dimensions. They further demonstrated that the proportion of PNECs in 2D hPSC-derived lung progenitor cultures treated with a Notch inhibitor could be further enriched by shRNA-mediated knockdown of *RB1*. Reduced levels of *RB1* expression resulted in the activation of a transcriptional program in PNECs that resembles that observed in human SCLC, indicating that this culture system might enable modelling of early stages of cancer development (Chen et al., 2019). Notch inhibition has also been used to derive PNECs from hPSCs in monolayer ALI cultures (Hor et al., 2020) and in airway organoids (Konishi et al., 2016). These models could inform our studies and understanding of the role of PNECs in respiratory diseases; however, we first need to better understand to what extent these derived PNECs resemble endogenous PNECs isolated from adult human lung tissue.

### Tissue stem cell-derived airway organoids

To date, several approaches have been used to generate airway organoids from tissue-resident, endogenous stem cells derived from adult or fetal lungs (van der Vaart and Clevers, 2021; Barkauskas et al., 2017). These systems offer a twofold benefit as they possess the self-renewal capacity of hPSCs and are less likely to generate off-target cell types, compared to cultures derived from hPSCs.

Until 2020, PNECs had not been reported in any airway organoids created from lung-derived pluripotent stem cells (Rock et al., 2009; Tan et al., 2017; Nikolić et al., 2017; Hild and Jaffe, 2016; Sachs et al., 2019). This was overcome by Miller et al. (2020) using SMAD signalling, followed by SMAD inhibition, to induce human bud tip progenitor differentiation, which created airway organoids that consisted of all the mature cell types of the proximal airways, including PNECs. The same group also investigated lineage relationships in the developing embryonic lung, applying a single-cell barcode-based lineage-tracing method to track the fate of progenitor cells during airway organoid differentiation *in vitro*. Their results support a model in which bud tip progenitors give rise to both LAP (see Introduction) and basal cells, which then give rise to distinct cell types. Notably, they observed that LAPs make a greater contribution to the generation of PNECs compared to that by basal cells in this airway organoid model (Conchola et al., 2023). Although these organoid systems can produce PNECs, the precise *in vitro* conditions responsible for driving the specification of lung progenitors into PNEC precursors and their subsequent maturation into differentiated PNECs remains unclear. Understanding these conditions could provide valuable insights into the lineage specification mechanisms guiding PNEC development during lung development. Strategies to efficiently obtain large numbers of PNECs in fetal and adult tissue-derived organoids could advance the study of this rare cell population in the future.

## Future perspectives

The intricate ways in which PNECs impact lung pathologies have only recently been understood and most remain largely unexplored. As discussed in this Review, a range of different *in vitro* airway models now provide us with platforms to investigate PNEC pathophysiology in human respiratory diseases. Below, we outline open questions relevant to the respiratory diseases discussed in this Review and propose how different model systems could be used to address these questions.

Understanding the mechanism by which PNECs are activated by known allergens in asthma and how they subsequently amplify immune responses could enable the design of PNEC-targeted therapies to dampen the aberrant airway inflammatory response observed in asthma. ALI culture systems containing PNECs could be leveraged to explore this question.

ALI culture systems could also be used to investigate the impact of volatile compounds and air pollutants on PNECs in the context of COPD. Such experiments could provide insights into the connection between the increase in exacerbation episodes in patients with COPD and their increased exposure to air pollutants. It would also be interesting to address the extent to which PNEC-secreted products, such as bombesins, contribute to the thickening of airways, leading to airflow obstruction in COPD. Exposing PCLSs to PNEC products may serve to decipher whether these products are sufficient to induce a COPD phenotype in the airways.

Given our limited knowledge regarding the involvement of PNECs in CF, deriving hiPSCs from patients with CF and using them to differentiate airway epithelia *in vitro* could enable us to investigate this further. For instance, it would be beneficial to investigate the effect of CFTR dysfunction on PNEC function and how this might contribute to the promotion of the pathological lung environment in CF.

There remains an unmet need for therapies specifically targeting lung NENs. Studying the dynamics of PNEC differentiation could contribute to resolving how lung NENs arise, and how they evolve into specific molecular subgroups and progress. A notable future breakthrough in this endeavour would be the successful generation of organoids containing PNECs from adult human tissue, a feat that has yet to be accomplished.

We predict that organoids derived from hPSCs or from tissue stem cells will significantly enhance our understanding of the pathophysiology of PNECs and advance the modelling of human respiratory diseases. Additionally, the implementation of state-of-the-art gene-editing techniques, such as CRISPR/Cas9, will enable researchers to genetically manipulate and label PNECs *in vitro* to facilitate their study. CRISPR/Cas9 could also be used, for example, to engineer organoid models of pulmonary cancers by introducing cancer-associated genetic alterations found in patients with lung cancer. The same approach could be used to introduce genetic variants that are linked (by genome-wide association studies) to an increased predisposition to respiratory diseases, such as asthma and COPD. Such an approach could assist in uncovering the molecular mechanisms that underlie these predispositions. It might also be possible to use this approach to obtain a better understanding of the factors that induce PNEC hyperplasia and pathology in patients with DIPNECH, by incorporating genetic variant candidates from familial DIPNECH into organoid models. Although airway organoids are valuable models, they are limited in their capacity to represent the complexity of the actual tissue as they lack crucial non-epithelial components. An intriguing, yet unexplored, strategy to address this limitation could be the co-culture of airway organoid-derived cells with PCLSs.

Our understanding of PNEC biology and the contribution of PNECS to respiratory diseases will be enhanced by the development of more precise *in vitro* models of the airway. As PNECs rely on interactions with non-epithelial cells, including with immune cells, neurons, endothelial cells and smooth muscle cells, there will be a need in the future to integrate these cellular components into existing models. As human airway epithelial models become more sophisticated, they will serve as valuable resources for advancing our understanding of PNEC biology. We predict that this progress will promote the development of new treatment strategies for respiratory diseases targeting PNECs or their secreted products.

## References

[DMM050620C1] Aguayo, S. M., Kane, M. A., King, T. E., Schwarz, M. I., Grauer, L. and Miller, Y. E. (1989). Increased levels of bombesin-like peptides in the lower respiratory tract of asymptomatic cigarette smokers. *J. Clin. Invest.* 84, 1105-1113. 10.1172/JCI114273 2794048 PMC329766

[DMM050620C2] Aguayo, S. M., King, T. E., Waldron, J. A., Sherritt, K. M., Kane, M. A. and Miller, Y. E. (1990). Increased pulmonary neuroendocrine cells with bombesin-like immunoreactivity in adult patients with eosinophilic granuloma. *J. Clin. Invest.* 86, 838-844. 10.1172/JCI114782 2394833 PMC296800

[DMM050620C3] Aguayo, S. M., Miller, Y. E., Waldron, J. A., Bogin, R. M., Sunday, M. E., Staton, G. W., Beam, W. R. and King, T. E. (1992). Idiopathic Diffuse Hyperplasia of Pulmonary Neuroendocrine Cells and Airways Disease. *N. Engl. J. Med.* 327, 1285-1288. 10.1056/NEJM199210293271806 1406819

[DMM050620C4] Al-Toubah, T., Strosberg, J., Halfdanarson, T. R., Oleinikov, K., Gross, D. J., Haider, M., Sonbol, M. B., Almquist, D. and Grozinsky-Glasberg, S. (2020). Somatostatin analogs improve respiratory symptoms in patients with diffuse idiopathic neuroendocrine cell hyperplasia. *Chest* 158, 401-405. 10.1016/j.chest.2020.01.031 32059961

[DMM050620C5] Alcala, N., Leblay, N., Gabriel, A. A. G., Mangiante, L., Hervas, D., Giffon, T., Sertier, A. S., Ferrari, A., Derks, J., Ghantous, A. et al. (2019). Integrative and comparative genomic analyses identify clinically relevant pulmonary carcinoid groups and unveil the supra-carcinoids. *Nat. Commun.* 10, 3407. 10.1038/s41467-019-11276-9 31431620 PMC6702229

[DMM050620C6] Baldassi, D., Gabold, B. and Merkel, O. M. (2021). Air-liquid interface cultures of the healthy and diseased human respiratory tract: promises, challenges, and future directions. *Adv. NanoBio. Res.* 1, 2000111. 10.1002/anbr.202000111 PMC7611446 34345878

[DMM050620C7] Barkauskas, C. E., Chung, M.-I., Fioret, B., Gao, X., Katsura, H. and Hogan, B. L. M. (2017). Lung organoids: current uses and future promise. *Development* 144, 986-997. 10.1242/dev.140103 28292845 PMC5358104

[DMM050620C8] Barnes, P. J., Burney, P. G. J., Silverman, E. K., Celli, B. R., Vestbo, J., Wedzicha, J. A. and Wouters, E. F. M. (2015). Chronic obstructive pulmonary disease. *Nat. Rev. Dis. Primers* 1, 15076. 10.1038/nrdp.2015.76 27189863

[DMM050620C9] Basil, M. C., Cardenas-Diaz, F. L., Kathiriya, J. J., Morley, M. P., Carl, J., Brumwell, A. N., Katzen, J., Slovik, K. J., Babu, A., Zhou, S. et al. (2022). Human distal airways contain a multipotent secretory cell that can regenerate alveoli. *Nature* 604, 120-126. 10.1038/s41586-022-04552-0 35355013 PMC9297319

[DMM050620C10] Bhogal, R., Sheldrick, R. L. G., Coleman, R. A., Smith, D. M. and Bloom, S. R. (1994). The effects of lAPP and CGRP on Guinea pig tracheal smooth muscle In Vitro. *Peptides* 15, 1243-1247. 10.1016/0196-9781(94)90148-1 7854976

[DMM050620C11] Boers, J. E., Den Brok, J. L., Koudstaal, J., Arends, J. W. and Thunnissen, F. B. (1996). Number and proliferation of neuroendocrine cells in normal human airway epithelium. *Am. J. Respir. Crit. Care. Med.* 154, 758-763. 10.1164/ajrccm.154.3.8810616 8810616

[DMM050620C12] Borges, M., Linnoila, R. I., van de Velde, H. J. K., Chen, H., Nelkin, B. D., Mabry, M., Baylin, S. B. and Ball, D. W. (1997). An achaete-scute homologue essential for neuroendocrine differentiation in the lung. *Nature* 386, 852-855. 10.1038/386852a0 9126746

[DMM050620C13] Branchfield, K., Nantie, L., Verheyden, J. M., Sui, P., Wienhold, M. D. and Sun, X. (2016). Pulmonary neuroendocrine cells function as airway sensors to control lung immune response. *Science* 351, 707-710. 10.1126/science.aad7969 26743624 PMC4860346

[DMM050620C14] Cardnell, R. J., Li, L., Sen, T., Bara, R., Tong, P., Fujimoto, J., Ireland, A. S., Guthrie, M. R., Bheddah, S., Banerjee, U. et al. (2017). Protein expression of TTF and cMYC define distinct molecular subgroups of small cell lung cancer with unique vulnerabilities to aurora kinase inhibition, DLL targeting, and other targeted therapies. *Oncotarget* 8, 73419-73432. 10.18632/oncotarget.20621 29088717 PMC5650272

[DMM050620C17] Chen, Y.-W., Huang, S. X., De Carvalho, A. L. R. T., Ho, S.-H., Islam, M. N., Volpi, S., Notarangelo, L. D., Ciancanelli, M., Casanova, J.-L., Bhattacharya, J. et al. (2017). A three-dimensional model of human lung development and disease from pluripotent stem cells. *Nat. Cell Biol.* 19, 542-549. 10.1038/ncb3510 28436965 PMC5777163

[DMM050620C18] Chen, H. J., Poran, A., Unni, A. M., Huang, S. X., Elemento, O., Snoeck, H.-W. and Varmus, H. (2019). Generation of pulmonary neuroendocrine cells and SCLC-like tumors from human embryonic stem cells. *J. Exp. Med.* 216, 674-687. 10.1084/jem.20181155 30737256 PMC6400536

[DMM050620C19] Conchola, A. S., Frum, T., Xiao, Z., Hsu, P. P., Kaur, K., Downey, M. S., Hein, R. F. C., Miller, A. J., Tsai, Y.-H., Wu, A. et al. (2023). Regionally distinct progenitor cells in the lower airway give rise to neuroendocrine and multiciliated cells in the developing human lung. *Proc. Natl Acad. Sci. USA*. 120, e2210113120. 10.1073/pnas.2210113120 37279279 PMC10268599

[DMM050620C20] Davies, S. J., Gosney, J. R., Hansell, D. M., Wells, A. U., du Bois, R. M., Burke, M. M., Sheppard, M. N. and Nicholson, A. G. (2007). Diffuse idiopathic pulmonary neuroendocrine cell hyperplasia: an under-recognised spectrum of disease. *Thorax* 62, 248-252. 10.1136/thx.2006.063065 17099078 PMC2117154

[DMM050620C22] De Proost, I., Pintelon, I., Brouns, I., Kroese, A. B. A., Riccardi, D., Kemp, P. J., Timmermans, J.-P. and Adriaensen, D. (2008). Functional live cell imaging of the pulmonary neuroepithelial body microenvironment. *Am. J. Respir. Cell Mol. Biol.* 39, 180-189. 10.1165/rcmb.2008-0011OC 18367726 PMC2643220

[DMM050620C23] Dye, B. R., Hill, D. R., Ferguson, M. A., Tsai, Y.-H., Nagy, M. S., Dyal, R., Wells, J. M., Mayhew, C. N., Nattiv, R., Klein, O. D. et al. (2015). In vitro generation of human pluripotent stem cell derived lung organoids. *Elife* 4, e05098. 25803487 10.7554/eLife.05098 PMC4370217

[DMM050620C24] Elborn, J. S. (2016). Cystic fibrosis. *Lancet* 388, 2519-2531. 10.1016/S0140-6736(16)00576-6 27140670

[DMM050620C25] Fernandez-Cuesta, L., Peifer, M., Lu, X., Sun, R., Ozretić, L., Seidel, D., Zander, T., Leenders, F., George, J., Müller, C. et al. (2014). Frequent mutations in chromatin-remodelling genes in pulmonary carcinoids. *Nat. Commun.* 5, 3518. 10.1038/ncomms4518 24670920 PMC4132974

[DMM050620C144] Ferone, G., Lee, M. C., Sage, J. and Berns, A. (2020). Cells of origin of lung cancers: lessons from mouse studies. *Genes Dev.* 34, 1017-1032. 10.1101/gad.338228.120 32747478 PMC7397855

[DMM050620C26] Feyrter, F. (1954). [Argyrophilia of bright cell system in bronchial tree in man]. *Z Mikrosk Anat Forsch* 61, 73-81. 14374964

[DMM050620C27] Firth, A. L., Dargitz, C. T., Qualls, S. J., Menon, T., Wright, R., Singer, O., Gage, F. H., Khanna, A. and Verma, I. M. (2014). Generation of multiciliated cells in functional airway epithelia from human induced pluripotent stem cells. *Proc. Natl. Acad. Sci. USA* 111, E1723-E1730. 10.1073/pnas.1403470111 24706852 PMC4035971

[DMM050620C28] Fisseler-Eckhoff, A. and Demes, M. (2012). Neuroendocrine tumors of the lung. *Cancers* 4, 777-798. 10.3390/cancers4030777 24213466 PMC3712715

[DMM050620C29] Flint, K., Ye, C. and Henry, T. L. (2019). Diffuse idiopathic pulmonary neuroendocrine cell hyperplasia (DIPNECH) with liver metastases. *BMJ Case Rep. CP* 12, e228536. 10.1136/bcr-2018-228536 PMC6605918 31239319

[DMM050620C30] Frohlich, F. (1949). [The light cell of the bronchial mucosa and its relationship to the problem of chemoreceptors]. *Frankf Z Pathol.* 60, 517-559. 18151654

[DMM050620C31] Fu, X. W., Nurse, C. A., Wang, Y. T. and Cutz, E. (1999). Selective modulation of membrane currents by hypoxia in intact airway chemoreceptors from neonatal rabbit. *J. Physiol.* 514, 139-150. 10.1111/j.1469-7793.1999.139af.x 9831722 PMC2269045

[DMM050620C32] Fu, X. W., Nurse, C. A., Wong, V. and Cutz, E. (2002). Hypoxia-induced secretion of serotonin from intact pulmonary neuroepithelial bodies in neonatal rabbit. *J. Physiol.* 539, 503-510. 10.1113/jphysiol.2001.013071 11882682 PMC2290169

[DMM050620C145] Garg, A., Sui, P., Verheyden, J. M., Young, L. R. and Sun, X. (2019). Consider the lung as a sensory organ: A tip from pulmonary neuroendocrine cells. *Curr. Top. Dev. Biol.* 132, 67-89. 10.1016/bs.ctdb.2018.12.002 30797518

[DMM050620C33] George, J., Lim, J. S., Jang, S. J., Cun, Y., Ozretić, L., Kong, G., Leenders, F., Lu, X., Fernández-Cuesta, L., Bosco, G. et al. (2015). Comprehensive genomic profiles of small cell lung cancer. *Nature* 524, 47-53. 10.1038/nature14664 26168399 PMC4861069

[DMM050620C34] George, J., Walter, V., Peifer, M., Alexandrov, L. B., Seidel, D., Leenders, F., Maas, L., Müller, C., Dahmen, I., Delhomme, T. M. et al. (2018). Integrative genomic profiling of large-cell neuroendocrine carcinomas reveals distinct subtypes of high-grade neuroendocrine lung tumors. *Nat. Commun.* 9, 1048. 10.1038/s41467-018-03099-x 29535388 PMC5849599

[DMM050620C35] Goldfarbmuren, K. C., Jackson, N. D., Sajuthi, S. P., Dyjack, N., Li, K. S., Rios, C. L., Plender, E. G., Montgomery, M. T., Everman, J. L., Bratcher, P. E. et al. (2020). Dissecting the cellular specificity of smoking effects and reconstructing lineages in the human airway epithelium. *Nat. Commun.* 11, 2485. 10.1038/s41467-020-16239-z 32427931 PMC7237663

[DMM050620C36] Goldsteen, P. A., Yoseif, C., Dolga, A. M. and Gosens, R. (2021). Human pluripotent stem cells for the modelling and treatment of respiratory diseases. *Eur. Respir. Rev.* 30, 210042. 10.1183/16000617.0042-2021 34348980 PMC9488746

[DMM050620C37] Gotoh, S., Ito, I., Nagasaki, T., Yamamoto, Y., Konishi, S., Korogi, Y., Matsumoto, H., Muro, S., Hirai, T., Funato, M. et al. (2014). Generation of alveolar epithelial spheroids via isolated progenitor cells from human pluripotent stem cells. *Stem Cell Rep.* 3, 394-403. 10.1016/j.stemcr.2014.07.005 PMC4266003 25241738

[DMM050620C38] Govindan, R., Page, N., Morgensztern, D., Read, W., Tierney, R., Vlahiotis, A., Spitznagel, E. L. and Piccirillo, J. (2006). Changing epidemiology of small-cell lung cancer in the United States over the last years: analysis of the surveillance, epidemiologic, and end results database. *J. Clin. Oncol.* 24, 4539-4544. 10.1200/JCO.2005.04.4859 17008692

[DMM050620C39] Green, M. D., Chen, A., Nostro, M.-C., d'Souza, S. L., Schaniel, C., Lemischka, I. R., Gouon-Evans, V., Keller, G. and Snoeck, H.-W. (2011). Generation of anterior foregut endoderm from human embryonic and induced pluripotent stem cells. *Nat. Biotechnol.* 29, 267-272. 10.1038/nbt.1788 21358635 PMC4866999

[DMM050620C40] Grozinsky-Glasberg, S., Shimon, I., Korbonits, M. and Grossman, A. B. (2008). Somatostatin analogues in the control of neuroendocrine tumours: efficacy and mechanisms. *Endocr. Relat. Cancer* 15, 701-720. 10.1677/ERC-07-0288 18524947

[DMM050620C41] Gu, X., Karp, P. H., Brody, S. L., Pierce, R. A., Welsh, M. J., Holtzman, M. J. and Ben-Shahar, Y. (2014). Chemosensory functions for pulmonary neuroendocrine cells. *Am. J. Respir. Cell Mol. Biol.* 50, 637-646. 10.1165/rcmb.2013-0199OC 24134460 PMC4068934

[DMM050620C42] Guha, A., Vasconcelos, M., Cai, Y., Yoneda, M., Hinds, A., Qian, J., Li, G., Dickel, L., Johnson, J. E., Kimura, S. et al. (2012). Neuroepithelial body microenvironment is a niche for a distinct subset of Clara-like precursors in the developing airways. *Proc. Natl. Acad. Sci. USA* 109, 12592-12597. 10.1073/pnas.1204710109 22797898 PMC3412014

[DMM050620C43] Guha, A., Deshpande, A., Jain, A., Sebastiani, P. and Cardoso, W. V. (2017). Uroplakin 3a+ cells are a distinctive population of epithelial progenitors that contribute to airway maintenance and post-injury repair. *Cell Rep.* 19, 246-254. 10.1016/j.celrep.2017.03.051 28402849

[DMM050620C44] Gunawardene, A. R., Corfe, B. M. and Staton, C. A. (2011). Classification and functions of enteroendocrine cells of the lower gastrointestinal tract. *Int. J. Exp. Pathol.* 92, 219-231. 10.1111/j.1365-2613.2011.00767.x 21518048 PMC3144510

[DMM050620C45] He, P., Lim, K., Sun, D., Pett, J. P., Jeng, Q., Polanski, K., Dong, Z., Bolt, L., Richardson, L., Mamanova, L. et al. (2022). A human fetal lung cell atlas uncovers proximal-distal gradients of differentiation and key regulators of epithelial fates. *Cell* 185, 4841-4860.e25. 10.1016/j.cell.2022.11.005 36493756 PMC7618435

[DMM050620C46] Hild, M. and Jaffe, A. B. (2016). Production of 3-D airway organoids from primary human airway basal cells and their use in high-throughput screening. *Curr. Protoc. Stem Cell Biol.* 37, IE.9.1-IE.9.15. 10.1002/cpsc.1 27171795

[DMM050620C47] Holgate, S. T. (2012). Innate and adaptive immune responses in asthma. *Nat. Med.* 18, 673-683. 10.1038/nm.2731 22561831

[DMM050620C48] Hong, K. U., Reynolds, S. D., Giangreco, A., Hurley, C. M. and Stripp, B. R. (2001). Clara cell secretory protein–expressing cells of the airway neuroepithelial body microenvironment include a label-retaining subset and are critical for epithelial renewal after progenitor cell depletion. *Am. J. Respir. Cell Mol. Biol.* 24, 671-681. 10.1165/ajrcmb.24.6.4498 11415931

[DMM050620C49] Hor, P., Punj, V., Calvert, B. A., Castaldi, A., Miller, A. J., Carraro, G., Stripp, B. R., Brody, S. L., Spence, J. R., Ichida, J. K. et al. (2020). Efficient generation and transcriptomic profiling of human iPSC-derived pulmonary neuroendocrine cells. *iScience* 23, 101083. 10.1016/j.isci.2020.101083 32380423 PMC7205764

[DMM050620C50] Huang, S. X. L., Islam, M. N., O'Neill, J., Hu, Z., Yang, Y.-G., Chen, Y.-W., Mumau, M., Green, M. D., Vunjak-Novakovic, G., Bhattacharya, J. et al. (2014). Efficient generation of lung and airway epithelial cells from human pluripotent stem cells. *Nat. Biotechnol.* 32, 84-91. 10.1038/nbt.2754 24291815 PMC4101921

[DMM050620C51] Huang, Y.-H., Klingbeil, O., He, X.-Y., Wu, X. S., Arun, G., Lu, B., Somerville, T. D. D., Milazzo, J. P., Wilkinson, J. E., Demerdash, O. E. et al. (2018). POU2F is a master regulator of a tuft cell-like variant of small cell lung cancer. *Genes Dev.* 32, 915-928. 10.1101/gad.314815.118 29945888 PMC6075037

[DMM050620C52] Ireland, A. S., Micinski, A. M., Kastner, D. W., Guo, B., Wait, S. J., Spainhower, K. B., Conley, C. C., Chen, O. S., Guthrie, M. R., Soltero, D. et al. (2020). MYC drives temporal evolution of small cell lung cancer subtypes by reprogramming neuroendocrine fate. *Cancer Cell* 38, 60-78.e12. 10.1016/j.ccell.2020.05.001 32473656 PMC7393942

[DMM050620C53] Ito, T., Udaka, N., Yazawa, T., Okudela, K., Hayashi, H., Sudo, T., Guillemot, F., Kageyama, R. and Kitamura, H. (2000). Basic helix-loop-helix transcription factors regulate the neuroendocrine differentiation of fetal mouse pulmonary epithelium. *Development* 127, 3913-3921. 10.1242/dev.127.18.3913 10952889

[DMM050620C54] Johnson, D. E., Wobken, J. D. and Landrum, B. G. (1988). Changes in bombesin, calcitonin, and serotonin immunoreactive pulmonary neuroendocrine cells in cystic fibrosis and after prolonged mechanical ventilation. *Am. Rev. Respir. Dis.* 137, 123-131. 10.1164/ajrccm/137.1.123 3337452

[DMM050620C55] Kadur Lakshminarasimha Murthy, P., Sontake, V., Tata, A., Kobayashi, Y., Macadlo, L., Okuda, K., Conchola, A. S., Nakano, S., Gregory, S., Miller, L. A. et al. (2022). Human distal lung maps and lineage hierarchies reveal a bipotent progenitor. *Nature* 604, 111-119. 10.1038/s41586-022-04541-3 35355018 PMC9169066

[DMM050620C56] Konishi, S., Gotoh, S., Tateishi, K., Yamamoto, Y., Korogi, Y., Nagasaki, T., Matsumoto, H., Muro, S., Hirai, T., Ito, I. et al. (2016). Directed induction of functional multi-ciliated cells in proximal airway epithelial spheroids from human pluripotent stem cells. *Stem Cell Rep.* 6, 18-25. 10.1016/j.stemcr.2015.11.010 PMC4720023 26724905

[DMM050620C57] Kuo, C. S. and Krasnow, M. A. (2015). Formation of a neurosensory organ by epithelial cell slithering. *Cell* 163, 394-405. 10.1016/j.cell.2015.09.021 26435104 PMC4597318

[DMM050620C58] Kuo, C. S., Darmanis, S., Diaz de Arce, A., Liu, A., Almanzar, Y., Wu, N., Quake, T. T.-H., Krasnow, S. R. and A, M. (2022). Neuroendocrinology of the lung revealed by single-cell RNA sequencing. *Elife* 11, e78216. 10.7554/eLife.78216 36469459 PMC9721618

[DMM050620C59] Lai, H. C., Meredith, D. M. and Johnson, J. E. (2013). bHLH factors in neurogenesis and neuronal subtype specification. In *Patterning and cell type specification in the developing CNS and PNS* (ed. J. L. R. Rubenstein and P. Rakic), pp. 333-354. Academic Press. 10.1016/B978-0-12-397265-1.00065-4

[DMM050620C60] Lambrecht, B. N. and Hammad, H. (2015). The immunology of asthma. *Nat. Immunol.* 16, 45-56. 10.1038/ni.3049 25521684

[DMM050620C61] Lauweryns, J. M. and Peuskens, J. C. (1972). Neuro-epithelial bodies (neuroreceptor or secretory organs?) in human infant bronchial and bronchiolar epithelium. *Anat. Rec.* 172, 471-481. 10.1002/ar.1091720301 4110997

[DMM050620C62] Lauweryns, J. M. and Van Lommel, A. (1987). Ultrastructure of nerve endings and synaptic junctions in rabbit intrapulmonary neuroepithelial bodies: a single and serial section analysis. *J. Anat.* 151, 65-83. 3654362 PMC1261701

[DMM050620C63] Lauweryns, J. M., Cokelaere, M., Deleersnyder, M. and Liebens, M. (1977). Intrapulmonary neuro-epithelial bodies in newborn rabbits. *Cell Tissue Res.* 182, 425-440. 10.1007/BF00219827 922815

[DMM050620C64] Lauweryns, J. M., Cokelaere, M., Lerut, T. and Theunynck, P. (1978). Cross-circulation studies on the influence of hypoxia and hypoxaemia on neuro-epithelial bodies in young rabbits. *Cell Tissue Res.* 193, 373-386. 10.1007/BF00225336 728949

[DMM050620C65] Lee, W., Lee, S., Yoon, J.-K., Lee, D., Kim, Y., Han, Y. B., Kim, R., Moon, S., Park, Y. J., Park, K. et al. (2023). A single-cell atlas of in vitro multiculture systems uncovers the in vivo lineage trajectory and cell state in the human lung. *Exp. Mol. Med.* 55, 1831-1842. 10.1038/s12276-023-01076-z 37582976 PMC10474282

[DMM050620C67] Liu, G., Betts, C., Cunoosamy, D. M., Åberg, P. M., Hornberg, J. J., Sivars, K. B. and Cohen, T. S. (2019). Use of precision cut lung slices as a translational model for the study of lung biology. *Respir. Res.* 20, 162. 10.1186/s12931-019-1131-x 31324219 PMC6642541

[DMM050620C69] Mathioudakis, A. G., Vanfleteren, L. E. G. W., Lahousse, L., Higham, A., Allinson, J. P., Gotera, C., Visca, D., Singh, D. and Spanevello, A. (2020). Current developments and future directions in COPD. *Eur. Respir. Rev.* 29, 200289. 10.1183/16000617.0289-2020 33268439 PMC9488623

[DMM050620C70] McCauley, K. B., Hawkins, F., Serra, M., Thomas, D. C., Jacob, A. and Kotton, D. N. (2017). Efficient derivation of functional human airway epithelium from pluripotent stem cells via temporal regulation of Wnt signaling. *Cell Stem Cell* 20, 844-857.e6. 10.1016/j.stem.2017.03.001 28366587 PMC5457392

[DMM050620C71] Miller, A. J., Yu, Q., Czerwinski, M., Tsai, Y.-H., Conway, R. F., Wu, A., Holloway, E. M., Walker, T., Glass, I. A., Treutlein, B. et al. (2020). In Vitro and In Vivo development of the human airway at single-cell resolution. *Dev. Cell* 53, 117-128.e6. 10.1016/j.devcel.2020.01.033 32109386 PMC7396815

[DMM050620C72] Modlin, I. M., Champaneria, M. C., Bornschein, J. and Kidd, M. (2006). Evolution of the diffuse neuroendocrine system – clear cells and cloudy origins. *Neuroendocrinology* 84, 69-82. 10.1159/000096997 17106184

[DMM050620C73] Mollaoglu, G., Guthrie, M. R., Böhm, S., Brägelmann, J., Can, I., Ballieu, P. M., Marx, A., George, J., Heinen, C., Chalishazar, M. D. et al. (2017). MYC drives progression of small cell lung cancer to a variant neuroendocrine subtype with vulnerability to aurora kinase inhibition. *Cancer Cell* 31, 270-285. 10.1016/j.ccell.2016.12.005 28089889 PMC5310991

[DMM050620C148] Montoro, D. T., Haber, A. L., Biton, M., Vinarsky, V., Lin, B., Birket, S. E., Yuan, F., Chen, S., Leung, H. M., Villoria, J. et al. (2018). A revised airway epithelial hierarchy includes CFTR-expressing ionocytes. *Nature* 560, 319-324. 10.1038/s41586-018-0393-7 30069044 PMC6295155

[DMM050620C74] Morimoto, M., Nishinakamura, R., Saga, Y. and Kopan, R. (2012). Different assemblies of Notch receptors coordinate the distribution of the major bronchial Clara, ciliated and neuroendocrine cells. *Development* 139, 4365-4373. 10.1242/dev.083840 23132245 PMC3509731

[DMM050620C75] Morrisey, E. E. and Hogan, B. L. M. (2010). Preparing for the first breath: genetic and cellular mechanisms in lung development. *Dev. Cell* 18, 8-23. 10.1016/j.devcel.2009.12.010 20152174 PMC3736813

[DMM050620C76] Mou, H., Zhao, R., Sherwood, R., Ahfeldt, T., Lapey, A., Wain, J., Sicilian, L., Izvolsky, K., Lau, F. H., Musunuru, K. et al. (2012). Generation of multipotent lung and airway progenitors from mouse ESCs and patient-specific cystic fibrosis iPSCs. *Cell Stem Cell* 10, 385-397. 10.1016/j.stem.2012.01.018 22482504 PMC3474327

[DMM050620C77] Mou, H., Yang, Y., Riehs, M. A., Barrios, J., Shivaraju, M., Haber, A. L., Montoro, D. T., Gilmore, K., Haas, E. A., Paunovic, B. et al. (2021). Airway basal stem cells generate distinct subpopulations of PNECs. *Cell Rep.* 35, 109011. 10.1016/j.celrep.2021.109011 33882306 PMC8140387

[DMM050620C78] Nikolić, M. Z., Caritg, O., Jeng, Q., Johnson, J.-A., Sun, D., Howell, K. J., Brady, J. L., Laresgoiti, U., Allen, G., Butler, R. et al. (2017). Human embryonic lung epithelial tips are multipotent progenitors that can be expanded in vitro as long-term self-renewing organoids. *Elife* 6, e26575. 10.7554/eLife.26575 28665271 PMC5555721

[DMM050620C79] Noguchi, M., Sumiyama, K. and Morimoto, M. (2015). Directed migration of pulmonary neuroendocrine cells toward airway branches organizes the stereotypic location of neuroepithelial bodies. *Cell Rep.* 13, 2679-2686. 10.1016/j.celrep.2015.11.058 26711336

[DMM050620C80] Noguchi, M., Furukawa, K. T. and Morimoto, M. (2020). Pulmonary neuroendocrine cells: physiology, tissue homeostasis and disease. *Dis. Model. Mech.* 13, dmm046920. 10.1242/dmm.046920 33355253 PMC7774893

[DMM050620C81] Okuda, K., Dang, H., Kobayashi, Y., Carraro, G., Nakano, S., Chen, G., Kato, T., Asakura, T., Gilmore, R. C., Morton, L. C. et al. (2021). Secretory cells dominate airway CFTR expression and function in human airway superficial epithelia. *Am. J. Respir. Crit. Care. Med.* 203, 1275-1289. 10.1164/rccm.202008-3198OC 33321047 PMC8456462

[DMM050620C83] Ouadah, Y., Rojas, E. R., Riordan, D. P., Capostagno, S., Kuo, C. S. and Krasnow, M. A. (2019). Rare pulmonary neuroendocrine cells are stem cells regulated by Rb, p53, and Notch. *Cell* 179, 403-416.e23. 10.1016/j.cell.2019.09.010 31585080 PMC6782070

[DMM050620C84] Pan, J., Luk, C., Kent, G., Cutz, E. and Yeger, H. (2006a). Pulmonary neuroendocrine cells, airway innervation, and smooth muscle are altered in Cftr null mice. *Am. J. Respir. Cell Mol. Biol.* 35, 320-326. 10.1165/rcmb.2005-0468OC 16614351 PMC2643285

[DMM050620C85] Pan, J., Copland, I., Post, M., Yeger, H. and Cutz, E. (2006b). Mechanical stretch-induced serotonin release from pulmonary neuroendocrine cells: implications for lung development. *Am. J. Physiol. Lung Cell. Mol. Physiol.* 290, L185-L193. 10.1152/ajplung.00167.2005 16100287

[DMM050620C86] Papi, A., Brightling, C., Pedersen, S. E. and Reddel, H. K. (2018). Asthma. *Lancet* 391, 783-800. 10.1016/S0140-6736(17)33311-1 29273246

[DMM050620C87] Park, K.-S., Liang, M.-C., Raiser, D. M., Zamponi, R., Roach, R. R., Curtis, S. J., Walton, Z., Schaffer, B. E., Roake, C. M., Zmoos, A.-F. et al. (2011). Characterization of the cell of origin for small cell lung cancer. *Cell Cycle* 10, 2806-2815. 10.4161/cc.10.16.17012 21822053 PMC3219544

[DMM050620C88] Placke, M. (1987). Adult peripheral lung organ culture—A model for respiratory tract toxicology. *Toxicol. Appl. Pharmacol.* 90, 284-298. 10.1016/0041-008X(87)90336-X 3629604

[DMM050620C89] Planer, J. D. and Morrisey, E. E. (2023). After the storm: regeneration, repair, and reestablishment of homeostasis between the alveolar epithelium and innate immune system following viral lung injury. *Annu. Rev. Pathol. Mech. Dis.* 18, 337-359. 10.1146/annurev-pathmechdis-031621-024344 PMC10875627 36270292

[DMM050620C90] Plasschaert, L. W., Žilionis, R., Choo-Wing, R., Savova, V., Knehr, J., Roma, G., Klein, A. M. and Jaffe, A. B. (2018). A single-cell atlas of the airway epithelium reveals the CFTR-rich pulmonary ionocyte. *Nature* 560, 377-381. 10.1038/s41586-018-0394-6 30069046 PMC6108322

[DMM050620C91] Plummer, H. K., III, Sheppard, B. J. and Schuller, H. M. (2000). Interaction of tobacco-specific toxicants with nicotinic cholinergic regulation of fetal pulmonary neuroendocrine cells: implications for pediatric lung disease. *Exp. Lung Res.* 26, 121-135. 10.1080/019021400269916 10742926

[DMM050620C92] Preuß, E. B., Schubert, S., Werlein, C., Stark, H., Braubach, P., Höfer, A., Plucinski, E. K. J., Shah, H. R., Geffers, R., Sewald, K. et al. (2022). The challenge of long-term cultivation of human precision-cut lung slices. *Am. J. Pathol.* 192, 239-253. 10.1016/j.ajpath.2021.10.020 34767811 PMC8891143

[DMM050620C93] Prieto, M., Chassagnon, G., Lupo, A., Charpentier, M.-C., Cabanne, E., Groussin, L., Wislez, M., Alifano, M. and Fournel, L. (2021). Lung carcinoid tumors with diffuse idiopathic pulmonary neuroendocrine cell hyperplasia (DIPNECH) exhibit pejorative pathological features. *Lung Cancer* 156, 117-121. 10.1016/j.lungcan.2021.04.024 33940544

[DMM050620C94] Ratcliffe, P., Pan, J., Bishop, T., Yeger, H. and Cutz, E. (2016). Hyperplasia and hypertrophy of pulmonary neuroepithelial bodies, presumed airway hypoxia sensors, in hypoxia-inducible factor prolyl hydroxylase-deficient mice. *Hypoxia (Auckl)* 4, 69-80. 10.2147/HP.S103957 27800509 PMC5085281

[DMM050620C95] Rawlins, E. L., Clark, C. P., Xue, Y. and Hogan, B. L. M. (2009). The Id2+ distal tip lung epithelium contains individual multipotent embryonic progenitor cells. *Development* 136, 3741-3745. 10.1242/dev.037317 19855016 PMC2766341

[DMM050620C96] Rekhtman, N. (2022). Lung neuroendocrine neoplasms: recent progress and persistent challenges. *Mod. Pathol.* 35, 36-50. 10.1038/s41379-021-00943-2 34663914 PMC8695375

[DMM050620C97] Reynolds, S. D., Giangreco, A., Power, J. H. T. and Stripp, B. R. (2000). Neuroepithelial bodies of pulmonary airways serve as a reservoir of progenitor cells capable of epithelial regeneration. *Am. J. Pathol.* 156, 269-278. 10.1016/S0002-9440(10)64727-X 10623675 PMC1868636

[DMM050620C98] Rindi, G., Klimstra, D. S., Abedi-Ardekani, B., Asa, S. L., Bosman, F. T., Brambilla, E., Busam, K. J., de Krijger, R. R., Dietel, M., El-Naggar, A. K. et al. (2018). A common classification framework for neuroendocrine neoplasms: an International Agency for Research on Cancer (IARC) and World Health Organization (WHO) expert consensus proposal. *Mod. Pathol.* 31, 1770-1786. 10.1038/s41379-018-0110-y 30140036 PMC6265262

[DMM050620C99] Riordan, J. R., Rommens, J. M., Kerem, B.-S., Alon, N., Rozmahel, R., Grzelczak, Z., Zielenski, J., Lok, S., Plavsic, N., Chou, J.-L. et al. (1989). Identification of the cystic fibrosis gene: cloning and characterization of complementary DNA. *Science* 245, 1066-1073. 10.1126/science.2475911 2475911

[DMM050620C100] Rock, J. R., Onaitis, M. W., Rawlins, E. L., Lu, Y., Clark, C. P., Xue, Y., Randell, S. H. and Hogan, B. L. M. (2009). Basal cells as stem cells of the mouse trachea and human airway epithelium. *Proc. Natl. Acad. Sci. USA* 106, 12771-12775. 10.1073/pnas.0906850106 19625615 PMC2714281

[DMM050620C101] Ross, A. J., Dailey, L. A., Brighton, L. E. and Devlin, R. B. (2007). Transcriptional profiling of mucociliary differentiation in human airway epithelial cells. *Am. J. Respir. Cell Mol. Biol.* 37, 169-185. 10.1165/rcmb.2006-0466OC 17413031

[DMM050620C102] Rossi, G., Cavazza, A., Spagnolo, P., Sverzellati, N., Longo, L., Jukna, A., Montanari, G., Carbonelli, C., Vincenzi, G., Bogina, G. et al. (2016). Diffuse idiopathic pulmonary neuroendocrine cell hyperplasia syndrome. *Eur. Respir. J.* 47, 1829-1841. 10.1183/13993003.01954-2015 27076588

[DMM050620C103] Röder, P. V., Wu, B., Liu, Y. and Han, W. (2016). Pancreatic regulation of glucose homeostasis. *Exp. Mol. Med.* 48, e219-e219. 10.1038/emm.2016.6 26964835 PMC4892884

[DMM050620C104] Sachs, N., Papaspyropoulos, A., Zomer-van Ommen, D. D., Heo, I., Böttinger, L., Klay, D., Weeber, F., Huelsz-Prince, G., Iakobachvili, N., Amatngalim, G. D. et al. (2019). Long-term expanding human airway organoids for disease modeling. *EMBO J.* 38, e100300. 10.15252/embj.2018100300 30643021 PMC6376275

[DMM050620C105] Saunders, L. R., Bankovich, A. J., Anderson, W. C., Aujay, M. A., Bheddah, S., Black, K., Desai, R., Escarpe, P. A., Hampl, J., Laysang, A. et al. (2015). A DLL3-targeted antibody-drug conjugate eradicates high-grade pulmonary neuroendocrine tumor-initiating cells in vivo. *Sci. Transl. Med.* 7, 302ra136. 10.1126/scitranslmed.aac9459 PMC4934375 26311731

[DMM050620C106] Shah, V. S., Chivukula, R. R., Lin, B., Waghray, A. and Rajagopal, J. (2022). Cystic fibrosis and the cells of the airway epithelium: what are ionocytes and what do they do? *Annu. Rev. Pathol. Mech. Dis.* 17, 23-46. 10.1146/annurev-pathol-042420-094031 PMC10837786 34437820

[DMM050620C107] Shenberger, J. S., Shew, R. L. and Johnson, D. E. (1997). Hyperoxia-induced airway remodeling and pulmonary neuroendocrine cell hyperplasia in the weanling rat. *Pediatr. Res.* 42, 539-544. 10.1203/00006450-199710000-00020 9380450

[DMM050620C108] Shivaraju, M., Chitta, U. K., Grange, R. M. H., Jain, I. H., Capen, D., Liao, L., Xu, J., Ichinose, F., Zapol, W. M., Mootha, V. K. et al. (2021). Airway stem cells sense hypoxia and differentiate into protective solitary neuroendocrine cells. *Science* 371, 52-57. 10.1126/science.aba0629 33384370 PMC8312065

[DMM050620C109] Song, H., Yao, E., Lin, C., Gacayan, R., Chen, M.-H. and Chuang, P.-T. (2012). Functional characterization of pulmonary neuroendocrine cells in lung development, injury, and tumorigenesis. *Proc. Natl. Acad. Sci. USA* 109, 17531-17536. 10.1073/pnas.1207238109 23047698 PMC3491514

[DMM050620C110] Sountoulidis, A., Marco Salas, S., Braun, E., Avenel, C., Bergenstråhle, J., Theelke, J., Vicari, M., Czarnewski, P., Liontos, A., Abalo, X. et al. (2023). A topographic atlas defines developmental origins of cell heterogeneity in the human embryonic lung. *Nat. Cell Biol.* 25, 351-365. 10.1038/s41556-022-01064-x 36646791 PMC9928586

[DMM050620C111] Stahlman, M. T., Jones, M., Gray, M. E., Kasselberg, A. G. and Vaughn, W. K. (1987). Ontogeny of neuroendocrine cells in human fetal lung III An electron microscopic immunohistochemical study. *Lab. Invest.* 56, 629-641. 3599909

[DMM050620C112] Stanislawski, E. C., Hernández-García, J., de la Mora-Torres, M. C. and Abraján-Polanco, E. (1981). Lung neuroendocrine structures Topography, morphology, composition and relation with intrinsic asthma (non-immune). *Arch. Invest. Med. (Mex)* 12, 559-577. 6120685

[DMM050620C113] Stewart, C. A., Gay, C. M., Xi, Y., Sivajothi, S., Sivakamasundari, V., Fujimoto, J., Bolisetty, M., Hartsfield, P. M., Balasubramaniyan, V., Chalishazar, M. D. et al. (2020). Single-cell analyses reveal increased intratumoral heterogeneity after the onset of therapy resistance in small-cell lung cancer. *Nat. Cancer* 1, 423-436. 10.1038/s43018-019-0020-z 33521652 PMC7842382

[DMM050620C114] Stupnikov, M. R., Yang, Y., Mori, M., Lu, J. and Cardoso, W. V. (2019). Jagged and Delta-like ligands control distinct events during airway progenitor cell differentiation. *Elife* 8, e50487. 10.7554/eLife.50487 31631837 PMC6887486

[DMM050620C115] Su, Y., Barr, J., Jaquish, A., Xu, J., Verheyden, J. M. and Sun, X. (2022). Identification of lung innervating sensory neurons and their target specificity. *Am. J. Physiol. Lung Cell. Mol. Physiol.* 322, L50-L63. 10.1152/ajplung.00376.2021 34755535 PMC8721910

[DMM050620C116] Su, Y., Xu, J., Zhu, Z., Yu, H., Nudell, V., Dash, B., Moya, E. A., Ye, L., Nimmerjahn, A. and Sun, X. (2023). Brainstem Dbh+ neurons control chronic allergen-induced airway hyperreactivity. *bioRxiv* 2023.02.04.527145. 10.1101/2023.02.04.527145 PMC11254774 38987587

[DMM050620C117] Sui, P., Wiesner, D. L., Xu, J., Zhang, Y., Lee, J., Van Dyken, S., Lashua, A., Yu, C., Klein, B. S., Locksley, R. M. et al. (2018). Pulmonary neuroendocrine cells amplify allergic asthma responses. *Science* 360, eaan8546. 10.1126/science.aan8546 29599193 PMC6387886

[DMM050620C118] Sunday, M. E. (1996). Pulmonary neuroendocrine cells and lung development. *Endocr Pathol.* 7, 173-201. 10.1007/BF02739921 12114731

[DMM050620C119] Sunday, M. E., Yoder, B. A., Cuttitta, F., Haley, K. J. and Emanuel, R. L. (1998). Bombesin-like peptide mediates lung injury in a baboon model of bronchopulmonary dysplasia. *J. Clin. Invest.* 102, 584-594. 10.1172/JCI2329 9691095 PMC508919

[DMM050620C120] Sutherland, K. D., Proost, N., Brouns, I., Adriaensen, D., Song, J.-Y. and Berns, A. (2011). Cell of origin of small cell lung cancer: inactivation of Trp and Rb in distinct cell types of adult mouse lung. *Cancer Cell* 19, 754-764. 10.1016/j.ccr.2011.04.019 21665149

[DMM050620C121] Tan, Q., Choi, K. M., Sicard, D. and Tschumperlin, D. J. (2017). Human airway organoid engineering as a step toward lung regeneration and disease modeling. *Biomaterials* 113, 118-132. 10.1016/j.biomaterials.2016.10.046 27815996 PMC5121055

[DMM050620C122] Temann, A., Golovina, T., Neuhaus, V., Thompson, C., Chichester, J. A., Braun, A. and Yusibov, V. (2017). Evaluation of inflammatory and immune responses in long-term cultured human precision-cut lung slices. *Hum. Vaccin. Immunother.* 13, 351-358. 10.1080/21645515.2017.1264794 27929748 PMC5328235

[DMM050620C123] Travaglini, K. J., Nabhan, A. N., Penland, L., Sinha, R., Gillich, A., Sit, R. V., Chang, S., Conley, S. D., Mori, Y., Seita, J. et al. (2020). A molecular cell atlas of the human lung from single-cell RNA sequencing. *Nature* 587, 619-625. 10.1038/s41586-020-2922-4 33208946 PMC7704697

[DMM050620C124] Travis, W. D. (2010). Advances in neuroendocrine lung tumors. *Ann. Oncol.* 21, vii65-vii71. 10.1093/annonc/mdq380 20943645

[DMM050620C125] Travis, W. D., Brambilla, E., Nicholson, A. G., Yatabe, Y., Austin, J. H. M., Beasley, M. B., Chirieac, L. R., Dacic, S., Duhig, E., Flieder, D. B. et al. (2015). The 201 World Health Organization classification of lung tumors: impact of genetic, clinical and radiologic advances since the 200 classification. *J. Thorac. Oncol.* 10, 1243-1260. 10.1097/JTO.0000000000000630 26291008

[DMM050620C126] Tränkner, D., Hahne, N., Sugino, K., Hoon, M. A. and Zuker, C. (2014). Population of sensory neurons essential for asthmatic hyperreactivity of inflamed airways. *Proc. Natl. Acad. Sci. USA* 111, 11515-11520. 10.1073/pnas.1411032111 25049382 PMC4128113

[DMM050620C127] van der Vaart, J. and Clevers, H. (2021). Airway organoids as models of human disease. *J. Intern. Med.* 289, 604-613. 10.1111/joim.13075 32350962

[DMM050620C128] Vos, T., Flaxman, A. D., Naghavi, M., Lozano, R., Michaud, C., Ezzati, M., Shibuya, K., Salomon, J. A., Abdalla, S., Aboyans, V. et al. (2012). Years lived with disability (YLDs) for 1160 sequelae of 289 diseases and injuries 1990–2010: a systematic analysis for the Global Burden of Disease Study 2010. *Lancet* 380, 2163-2196. 10.1016/S0140-6736(12)61729-2 23245607 PMC6350784

[DMM050620C129] Vos, T., Lim, S. S., Abbafati, C., Abbas, K. M., Abbasi, M., Abbasifard, M., Abbasi-Kangevari, M., Abbastabar, H., Abd-Allah, F., Abdelalim, A. et al. (2020). Global burden of 369 diseases and injuries in 204 countries and territories, 1990–2019: a systematic analysis for the Global Burden of Disease Study 2019. *Lancet* 396, 1204-1222. 10.1016/S0140-6736(20)30925-9 33069326 PMC7567026

[DMM050620C147] Waghray, A., Monga, I., Lin, B., Shah, V., Slyper, M., Giotti, B., Xu, J., Waldman, J., Dionne, D., Nguyen, L. T. et al. (2023). A deep lung cell atlas reveals cytokine-mediated lineage switching of a rare cell progenitor of the human airway epithelium. *bioRxiv* 2023.11.28.569028. 10.1101/2023.11.28.569028

[DMM050620C131] Whitcutt, M. J., Adler, K. B. and Wu, R. (1988). A biphasic chamber system for maintaining polarity of differentiation of cultured respiratory tract epithelial cells. *In Vitro Cell. Dev. Biol.* 24, 420-428. 10.1007/BF02628493 3372447

[DMM050620C132] Willey, J. C., Lechner, J. F. and Harris, C. C. (1984). Bombesin and the C-terminal tetradecapeptide of gastrin-releasing peptide are growth factors for normal human bronchial epithelial cells. *Exp. Cell Res.* 153, 245-248. 10.1016/0014-4827(84)90466-X 6547391

[DMM050620C133] Wong, A. P., Bear, C. E., Chin, S., Pasceri, P., Thompson, T. O., Huan, L.-J., Ratjen, F., Ellis, J. and Rossant, J. (2012). Directed differentiation of human pluripotent stem cells into mature airway epithelia expressing functional CFTR protein. *Nat. Biotechnol.* 30, 876-882. 10.1038/nbt.2328 22922672 PMC3994104

[DMM050620C134] Xu, H., Zhao, M. and Wang, X. (1999). [Changes of calcitonin gene-related peptide content in induced sputum from patients with COPD and asthma]. *Zhonghua Jie He He Hu Xi Za Zhi* 22, 558-561. 11776772

[DMM050620C135] Xu, J., Yu, H. and Sun, X. (2020). Less is more: rare pulmonary neuroendocrine cells function as critical sensors in lung. *Dev. Cell* 55, 123-132. 10.1016/j.devcel.2020.09.024 33108755

[DMM050620C136] Yamamoto, Y., Gotoh, S., Korogi, Y., Seki, M., Konishi, S., Ikeo, S., Sone, N., Nagasaki, T., Matsumoto, H., Muro, S. et al. (2017). Long-term expansion of alveolar stem cells derived from human iPS cells in organoids. *Nat. Methods* 14, 1097-1106. 10.1038/nmeth.4448 28967890

[DMM050620C137] Yamaya, M., Finkbeiner, W. E., Chun, S. Y. and Widdicombe, J. H. (1992). Differentiated structure and function of cultures from human tracheal epithelium. *Am. J. Physiol. Lung Cell. Mol. Physiol.* 262, L713-L724. 10.1152/ajplung.1992.262.6.L713 1616056

[DMM050620C138] Yang, D., Denny, S. K., Greenside, P. G., Chaikovsky, A. C., Brady, J. J., Ouadah, Y., Granja, J. M., Jahchan, N. S., Lim, J. S., Kwok, S. et al. (2018a). Intertumoral heterogeneity in SCLC is influenced by the cell type of origin. *Cancer Discov.* 8, 1316-1331. 10.1158/2159-8290.CD-17-0987 30228179 PMC6195211

[DMM050620C139] Yang, Y., Riccio, P., Schotsaert, M., Mori, M., Lu, J., Lee, D.-K., García-Sastre, A., Xu, J. and Cardoso, W. V. (2018b). Spatial-temporal lineage restrictions of embryonic p63+ progenitors establish distinct stem cell pools in adult airways. *Dev. Cell* 44, 752-761e4. 10.1016/j.devcel.2018.03.001 29587145 PMC5875454

[DMM050620C140] Yoon, J. Y., Sigel, K., Martin, J., Jordan, R., Beasley, M. B., Smith, C., Kaufman, A., Wisnivesky, J. and Kim, M. K. (2019). Evaluation of the prognostic significance of TNM staging guidelines in lung carcinoid tumors. *J. Thorac. Oncol.* 14, 184-192. 10.1016/j.jtho.2018.10.166 30414942

[DMM050620C141] Youngson, C., Nurse, C., Yeger, H. and Cutz, E. (1993). Oxygen sensing in airway chemoreceptors. *Nature* 365, 153-155. 10.1038/365153a0 8371757

[DMM050620C142] Yu, W., Moninger, T. O., Rector, M. V., Stoltz, D. A. and Welsh, M. J. (2022a). Pulmonary neuroendocrine cells sense succinate to stimulate myoepithelial cell contraction. *Dev. Cell* 57, 2221-2236.e5. 10.1016/j.devcel.2022.08.010 36108628 PMC9762774

[DMM050620C143] Yu, W., Moninger, T. O., Thurman, A. L., Xie, Y., Jain, A., Zarei, K., Powers, L. S., Pezzulo, A. A., Stoltz, D. A. and Welsh, M. J. (2022b). Cellular and molecular architecture of submucosal glands in wild-type and cystic fibrosis pigs. *Proc. Natl. Acad. Sci. USA* 119, e2119759119. 10.1073/pnas.2119759119 35046051 PMC8794846

